# Aromatic ^19^F–^13^C TROSY—[^19^F, ^13^C]‐Pyrimidine Labeling for NMR Spectroscopy of RNA

**DOI:** 10.1002/anie.202006577

**Published:** 2020-07-29

**Authors:** Felix Nußbaumer, Raphael Plangger, Manuel Roeck, Christoph Kreutz

**Affiliations:** ^1^ Institute of Organic Chemistry and Center for Molecular Biosciences Innsbruck (CMBI) University of Innsbruck Innrain 80/82 6020 Innsbruck Austria

**Keywords:** 19F-NMR, RNA, stable isotope labeling, TROSY

## Abstract

We present the access to [5‐^19^F, 5‐^13^C]‐uridine and ‐cytidine phosphoramidites for the production of site‐specifically modified RNAs up to 65 nucleotides (nts). The amidites were used to introduce [5‐^19^F, 5‐^13^C]‐pyrimidine labels into five RNAs—the 30 nt human immunodeficiency virus *trans* activation response (HIV TAR) 2 RNA, the 61 nt human hepatitis B virus *ϵ* (hHBV *ϵ*) RNA, the 49 nt SAM VI riboswitch aptamer domain from *B. angulatum*, the 29 nt apical stem loop of the pre‐microRNA (miRNA) 21 and the 59 nt full length pre‐miRNA 21. The main stimulus to introduce the aromatic ^19^F–^13^C‐spin topology into RNA comes from a work of Boeszoermenyi et al., in which the dipole‐dipole interaction and the chemical shift anisotropy relaxation mechanisms cancel each other leading to advantageous TROSY properties shown for aromatic protein sidechains. This aromatic ^13^C–^19^F labeling scheme is now transferred to RNA. We provide a protocol for the resonance assignment by solid phase synthesis based on diluted [5‐^19^F, 5‐^13^C]/[5‐^19^F] pyrimidine labeling. For the 61 nt hHBV *ϵ* we find a beneficial ^19^F–^13^C TROSY enhancement, which should be even more pronounced in larger RNAs and will facilitate the NMR studies of larger RNAs. The [^19^F, ^13^C]‐labeling of the SAM VI aptamer domain and the pre‐miRNA 21 further opens the possibility to use the biorthogonal stable isotope reporter nuclei in in vivo NMR to observe ligand binding and microRNA processing in a biological relevant setting.

## Graphical abstract

^**19**^ **F and** ^**13**^ **C in RNA—a perfect match**. An aromatic ^19^F–^13^C isotope labeling protocol is introduced for uridines and cytidines in RNA up to 60 nucleotides. The favorable TROSY properties of this spin pair will be useful to probe large RNAs by NMR spectroscopy.

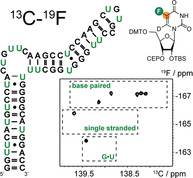

## Introduction

^19^F‐NMR is a well‐recognized technique to study biomolecules. The fluorine nucleus offers a 100 % natural abundance, a high sensitivity, a larger chemical shift dispersion than protons, and is biorthogonal making it an ideal candidate for in vivo NMR applications. Thus, it is not surprising that several ^19^F‐NMR spectroscopic studies to probe structure/function of nucleic acids can be found in literature. Fluorinated nucleotides, such as sugar fluorinated residues,^1^ 5‐F‐uridines and ‐cytidines,^2^ 2‐F‐adenosines,^3^ but also more complex modifications,^4^ were incorporated in RNAs up to 70 nts. The fluorine labels were used to address RNA secondary and tertiary structure heterogeneity, folding kinetics, protein and small molecule binding and recently to detect G‐quadruplexes in living cells by NMR.^5^ The approaches relied either on solid phase synthesis of the nucleic acid to introduce fluorinated residues at freely selectable positions thereby circumventing issues regarding the resonance assignment or on enzymatic RNA production. Schwalbe and co‐workers used the latter method to probe the effect of all 2‐F‐A labeling in the 73 nt guanine sensing riboswitch from *bacillus subtilis*.3a Only four resonances of sixteen could be assigned illustrating that in larger RNAs resonance assignment issues arise. Thus, an expansion of the ^19^F stable isotope labeling scheme to yield a ^19^F–^13^C‐spin topology, such as a [5‐^19^F, 5‐^13^C]‐labeling pattern in pyrimidines, would be desirable to evade resonance overlap issues in larger RNAs by adding a second ^13^C dimension. The application of ^19^F‐NMR to study high molecular weight systems is also hampered by the fast transverse relaxation by the chemical shift anisotropy (CSA) mechanism. In a recent work this issue was addressed by introducing aromatic ^19^F–^13^C‐spin topologies in proteins and DNA, in which the cancellation of the dipole‐dipole interaction and the chemical shift anisotropy relaxation mechanisms lead to a very favorable NMR spectroscopic behavior in a TROSY type experiment.^6^ Here, we report on the synthetic access to [5‐^19^F, 5‐^13^C]‐uridine (U) and ‐cytidine (C) phosphoramidites for solid phase RNA synthesis. We demonstrate the favorable spectroscopic behavior of the [5‐^19^F, 5‐^13^C]‐pyrimidines in two model RNAs: the 61 nt human hepatitis B virus encapsidation signal epsilon (hHBV *ϵ*) and the 59 nt pre‐microRNA 21.^7^ The feasibility of the ^13^C/^19^F labeling by solid phase RNA synthesis is shown and used for a resonance assignment protocol based on [5‐^19^F, 5‐^13^C]/[5‐^19^F]‐uridine labeling schemes. For the 61 nt hHBV *ϵ* RNA we observed a favorable TROSY effect and compared the performance of [5‐^19^F, ^13^C]‐U labels with [6‐^1^H, 6‐^13^C]‐U labels. The novel stable isotope (SI) labeling scheme will facilitate NMR studies of large RNAs and high molecular weight RNA protein complexes as it was recently demonstrated for high molecular weight proteins systems.^6^ Combining the approach with existing ligation strategies will also allow to put a spotlight on structural and functional features in distinct regions of large RNAs.^8^

## Results and Discussion

### The Aromatic RNA ^19^F–^13^C‐Labeling Strategy and the Sequences Used in this Study

**Figure 1:**
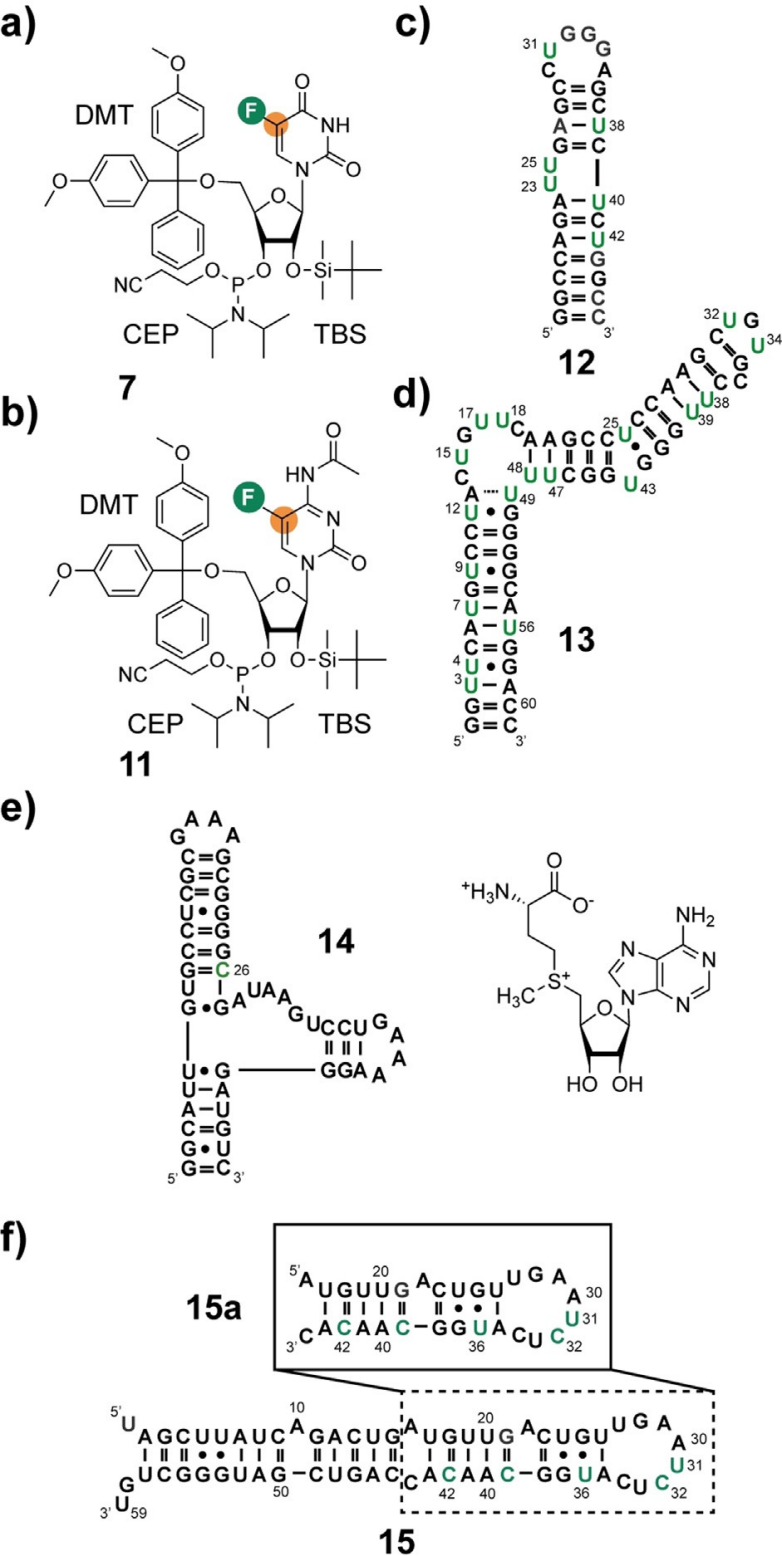
[5‐^19^F, 5‐^13^C]‐pyrimidine RNA building blocks and target RNAs. a) [5‐^19^F, 5‐^13^C]‐uridine and b) [5‐^19^F, 5‐^13^C]‐cytidine phosphoramidite building blocks **7** and **11** suitable for RNA production via solid phase synthesis. c) HIV TAR 2 RNA **12** with 30 nucleotides (nts) and a molecular weight of approx. 10 kD. d) hHBV *ϵ* RNA **13** with 61 nts and a molecular weight of approx. 20 kD. e) SAM VI *metK* aptamer domain **14** with 49 nts, the *S*‐adenosyl‐l‐methionine ligand is shown. f) The 59 nt pre‐miRNA 21 **15** and the apical stem loop subsegment **15 a**. Fluorine is highlighted in green, orange dot=^13^C, [^19^F,^13^C]‐modified residues highlighted in green.

Based on the TROSY calculations by Boeszoermenyi et al. and building upon a previously established synthetic route to introduce pyrimidine ^13^C5 labels we synthesized the [5‐^19^F, 5‐^13^C]‐uridine and ‐cytidine building blocks **7** and **11** (Figure 1 a and b).^6^, ^9^ Additionally, a [6‐D, 5‐^19^F, 5‐^13^C]‐uridine RNA phosphoramidite was produced to minimize the scalar coupling interactions and to give optimal results on probes without the option to decouple protons. To test the incorporation of the [5‐^19^F, 5‐^13^C]‐U label by solid phase RNA synthesis, we picked the HIV‐TAR 2 RNA **12** with six uridines. This RNA was previously studied by ^19^F‐NMR by Hennig and co‐workers (Figure 1 c).2c To investigate the favorable ^19^F–^13^C‐TROSY effect we introduce the [5‐^19^F, 5‐^13^C]‐U labels into the larger human hepatitis B virus encapsidation (hHBV *ϵ*) RNA **13** with 61 nts (ca. 20 kD) (Figure 1 d). We further incorporated a [5‐^19^F, 5‐^13^C]‐C label at position 26 into the 49 nt SAM VI aptamer **14** from *B. angulatum* to monitor the ligand binding event in a biologically relevant riboswitch (Figure 1 e). Finally, we combined [5‐^19^F, 5‐^13^C]‐C and ‐U labeling in two variants of the microRNA 21. RNA **15** is the 59 nt oncogenic pre‐microRNA (miRNA) 21 after processing by the microprocessor enzyme complex and carries three [5‐^19^F, 5‐^13^C]‐cytidine (C32, C39, C42) and two [6‐D, 5‐^19^F, 5‐^13^C]‐uridine (U31, U36) labels. Further, the apical stem loop **15 a** with the same labeling pattern was produced to probe the modular composition of this pre‐miRNA 21 (Figure 1 f).

### Synthetic Access to the [5‐^19^F, 5‐^13^C]‐Uridine and ‐Cytidine Phosphoramidite 7 and 11

**Scheme 1:**
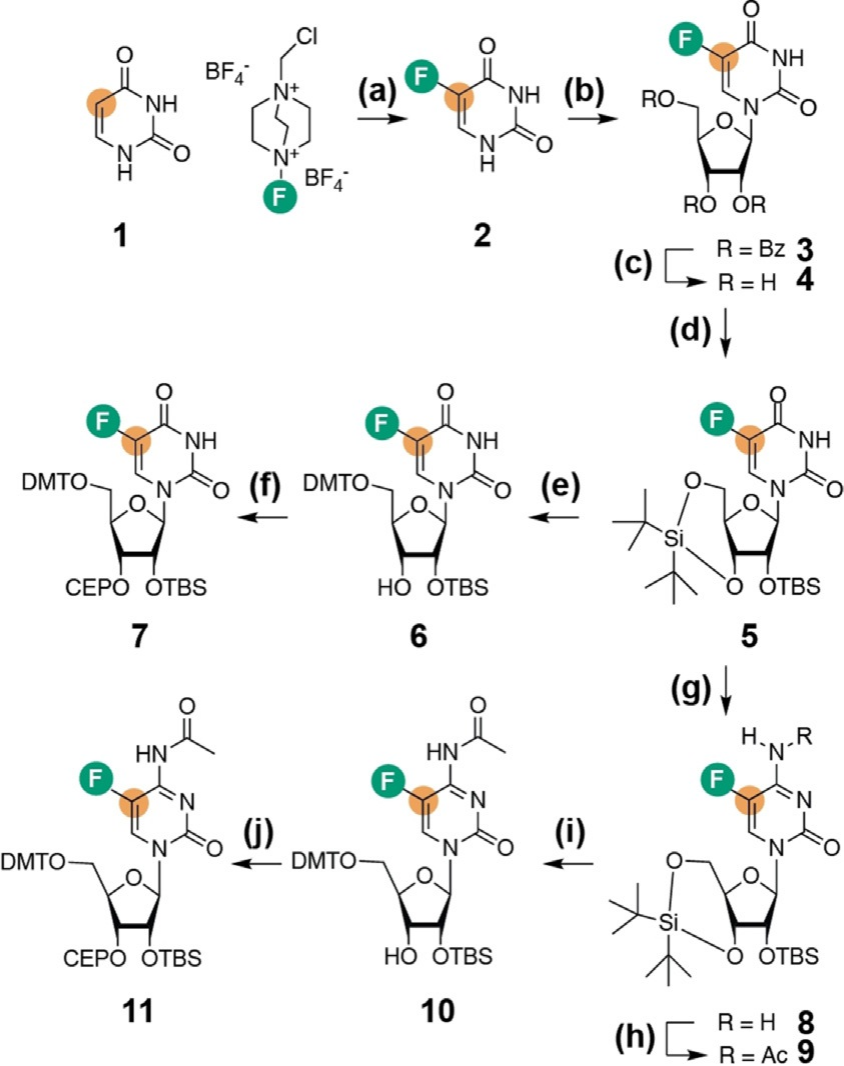
Synthetic access to the phosphoramidites **7** and **11**. a) F‐TEDA (Selectfluor^TM^) in water, 90 °C, 16 h, 47 %; b) 1‐*O*‐Acetyl‐2,3,5‐tri‐*O*‐benzoyl‐β‐d‐ribofuranose, *N*,*O*‐bis(trimethylsilyl)acetamide, trimethylsilyl trifluoromethane sulfonate in absolute acetonitrile, 60 °C, 1 h, 64 %; c) methylamine in ethanol (33 wt %), rt, 16 h, 88 %; d) di‐*tert*‐butylsilyl bis(trifluoromethanesulfonate) in absolute DMF, 0 °C, 1 h, then imidazole, *tert*‐butyl dimethylsilyl chloride, 60 °C, 2 h, 70 %; e) HF‐pyridine in methylene chloride, 0 °C, 2 h, then 4,4′‐dimethoxytrityl chloride in pyridine, rt, 3 h, 47 %; f) 2‐Cyanoethyl *N*,*N*,*N*′,*N*′‐tetraisopropylphosphorodiamidite, 5‐(benzylthio)‐1*H*‐tetrazole in absolute acetonitrile, rt, 16 h, 89 %. g) 2,4,6‐triisopropylbenzenesulfonyl chloride, triethylamine, dimethylaminopyridine in anhydrous acetonitrile, then 28 % aqueous ammonia, 0 °C to rt, 20 h, 62 %; h) Acetic anhydride in absolute DMF, rt, 16 h, 74 %; i) HF‐pyridine in methylene chloride, 0 °C, 2 h, then 4,4′‐dimethoxytrityl chloride in pyridine, rt, 3 h, 64 %; j) 2‐Cyanoethyl *N*,*N*,*N*′,*N*′‐tetraisopropylphosphorodiamidite, 5‐(benzylthio)‐1*H*‐tetrazole in absolute acetonitrile, rt, 16 h, 70 %. Fluorine is highlighted in green, ^13^C=orange dot.

Our initial efforts focused on the production of the [5‐^19^F, 5‐^13^C]‐uridine phosphoramidite **7**. Based on our recently published synthetic access to a 5‐^13^C‐uridine building block^9^ we included a fluorination step using the commercially available fluorination reagent Selectfluor^TM^. The synthetic route starting from [5‐^13^C]‐uracil is shown (Scheme 1). The compound **1** was produced from [2‐^13^C]‐bromoacetic acid, potassium cyanide and urea as previously described. The key step to introduce the aromatic ^19^F–^13^C spin topology was the pyrimidine position 5 fluorination using the electrophilic *Selectfluor* reagent with ca. 50–60 % yield of compound **2**. In the next step benzoylated nucleoside **3** was obtained via a silyl‐Hilbert‐Johnson nucleosidation reaction, which was deprotected under alkaline conditions to give **4**. At this stage position 6 can be deuterated by treating **4** with sodium deuteroxide in D_2_O for 3 h under reflux. The final steps included a transient 5′‐, 3′‐hydroxyl protection to allow the regioselective introduction of the 2′‐*O*‐*tert*‐butyldimethyl silyl (TBS) group, followed by the 5′‐tritylation reaction to give compound **6**. Finally, the [5‐^19^F, 5‐^13^C]‐uridine 3′‐phosphoramidite **7** was obtained. Noteworthy, we found it crucial to substitute triethylamine by pyridine as the additive for the eluents used in the silica chromatography to get reproducible recovery rates after column chromatography. Phosphoramidite **7** could be obtained in a six‐step synthesis with an overall yield of 8 %. Using the protected **5** the [5‐^19^F, 5‐^13^C]‐cytidine 3′‐phosphoramidite **11** could be obtained by transforming the uridine nucleoside into the respective cytidine derivative **8** followed by an exocyclic amino selective acetylation step yielding **9**. The final steps included the removal of the transient 5′‐, 3′‐hydroxy protection, followed by the 5′‐tritylation to give compound **10**. Finally, the [5‐^19^F, 5‐^13^C]‐cytidine 3′‐phosphoramidite **11** was obtained via the phosphitylation reaction with a yield of 70 %. We observed a 30 % proton back‐exchange of the deuterium at position 6 during the uridine to cytidine transformation using aqueous ammonia in acetonitrile at room temperature. Thus, we decided to produce only building block **11**, as we presume that during the strongly alkaline deprotection at elevated temperatures in the oligonucleotide synthesis a mixture of [6‐D/H, 5‐^19^F, 5‐^13^C]‐cytidine will be obtained. Both amidites were then used in the solid phase synthesis approach to label RNAs up to 60 nts. A short discussion on the aspects of chemical solid phase synthesis of [5‐^19^F, 5‐^13^C]‐pyrimidine modified RNAs is given in the supporting information.

### ^13^C‐Detected Out‐and‐Stay ^19^F–^13^C‐TROSY Experiments of [5‐^19^F, 5‐^13^C]‐Uridine Labeled RNAs 12 and 13

**Figure 2:**
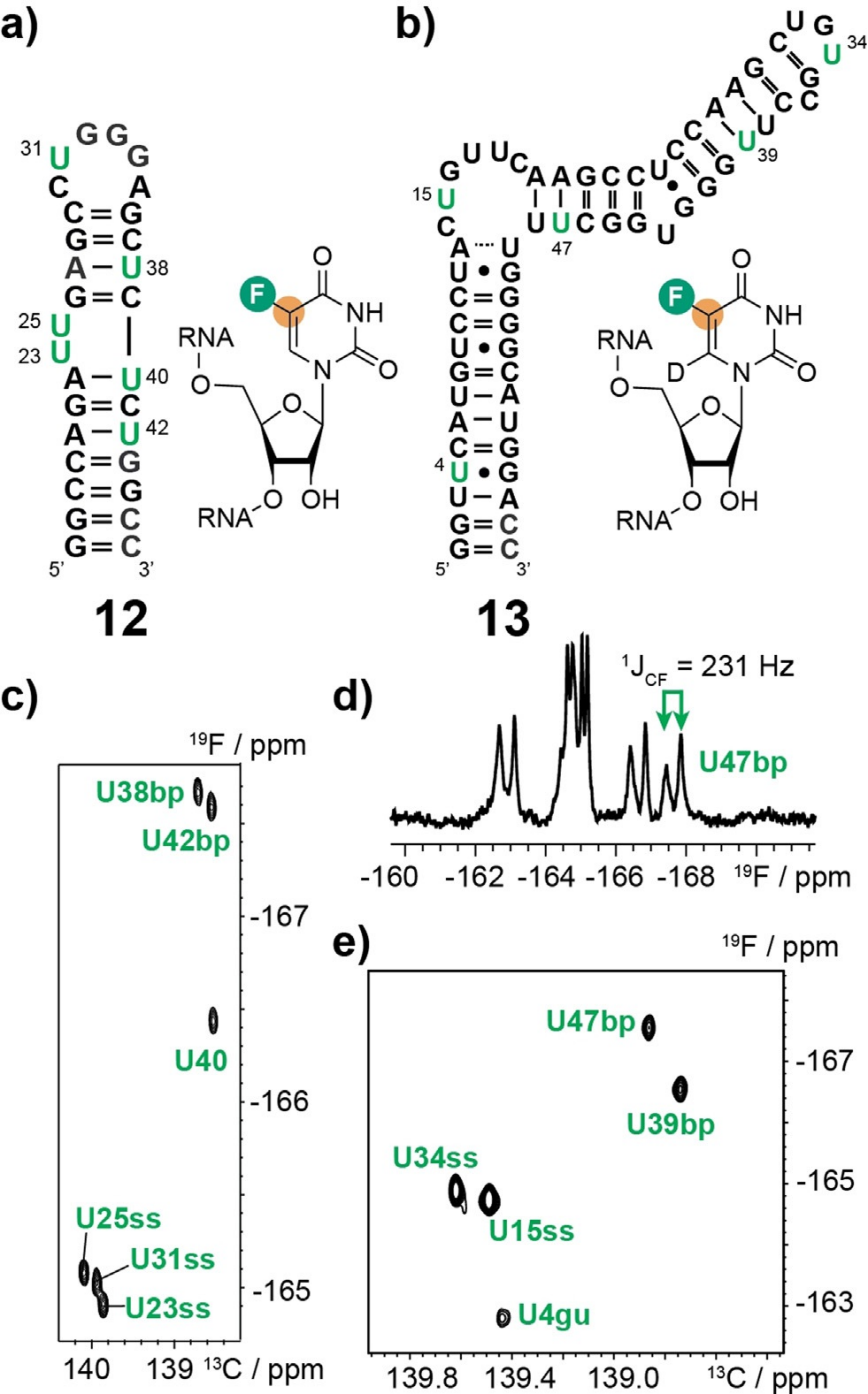
^13^C‐detected out‐and‐stay ^19^F–^13^C‐TROSY of [5‐^19^F, 5‐^13^C]‐uridine labeled RNAs **12** and **13**. a) Secondary structure of RNA **12** with the [5‐^19^F, 5‐^13^C]‐uridine labels highlighted in green. The inset shows the [5‐^19^F, 5‐^13^C]‐uridine label. b) Secondary structure of the hHBV *ϵ* RNA with the [6‐D, 5‐^19^F, 5‐^13^C]‐uridine labels highlighted in green. The inset shows the [6‐D, 5‐^19^F, 5‐^13^C]‐uridine label. c) ^13^C‐detected out‐and‐stay ^19^F–^13^C‐TROSY of **12** with assignments. d) ^19^F‐NMR spectrum of RNA **13** without ^13^C decoupling. The U47 resonance is highlighted and the coupling constant ^1^ *J*_CF_ is given. e) ^13^C‐detected out‐and‐stay ^19^F–^13^C‐TROSY of RNA **13** with assignments.

As the first target, the 30 nt HIV TAR‐2 RNA was chosen as for this system ^19^F NMR assignment data for an in vitro transcribed [5‐^19^F]‐uridine labeled version is available.2c All six uridines were substituted by the [5‐^19^F, 5‐^13^C]‐counterparts (Figure 2 a). We decided to address [5‐^19^F, 5‐^13^C]‐stable isotope labeling in the larger hHBV *ϵ* RNA with 61 nts with a molecular weight of ≈20 kD. In the first construct five uridines—U4, U15, U34 and U47—were replaced by their [6‐D, 5‐^19^F, 5‐^13^C]‐counterparts (Figure 2 b). As reported by Arthanari and co‐workers the ^13^C‐ detected out‐and‐stay ^19^F–^13^C‐TROSY gave the best results (Figure 2 c and e).^6^ The ^13^C‐detected TROSYs were acquired on a 600 MHz NMR with a TCI Prodigy probe with the proton coil tuned to ^19^F. Each ^19^F–^13^C correlation spectrum was recorded within 1 h using 0.2–0.5 mm samples. The assignments of **12** could be transferred from the work by Hennig and co‐workers. The resonances of **13** were assigned by site‐specific labeling. The ^19^F‐NMR spectrum without ^13^C decoupling of **13** qualitatively reflects the favorable TROSY effect in the ^19^F/^13^C spin system illustrated by the asymmetric doublet intensities and the sharper line width of the high field resonance of U47 (Figure 2 d). We found a fold specific ^19^F‐chemical shift distribution in both RNAs **12** and **13**. For RNA **12** the Watson–Crick base paired U38 and U42 ^19^F resonances clustered at −167 ppm, whereas the single stranded U23, U25, and U31 signals were clustered around −165 ppm. U40 with a ^19^F chemical shift of −166.4 ppm fluctuates between a single stranded and a base paired conformation leading to this intermediate ^19^F resonance around −166 ppm. The same shift distribution was found for RNA **13** with base paired uridines U47 and U39 at −167 ppm and single stranded uridine fluorine peak positions at −165 ppm. We further found a G–U wobble base pair specific ^19^F chemical shift of U4 at −163 ppm.

### Assignment of All [5‐^19^F, 5‐^13^C]‐Uridine hHBV *ϵ* RNA Resonances

**Figure 3:**
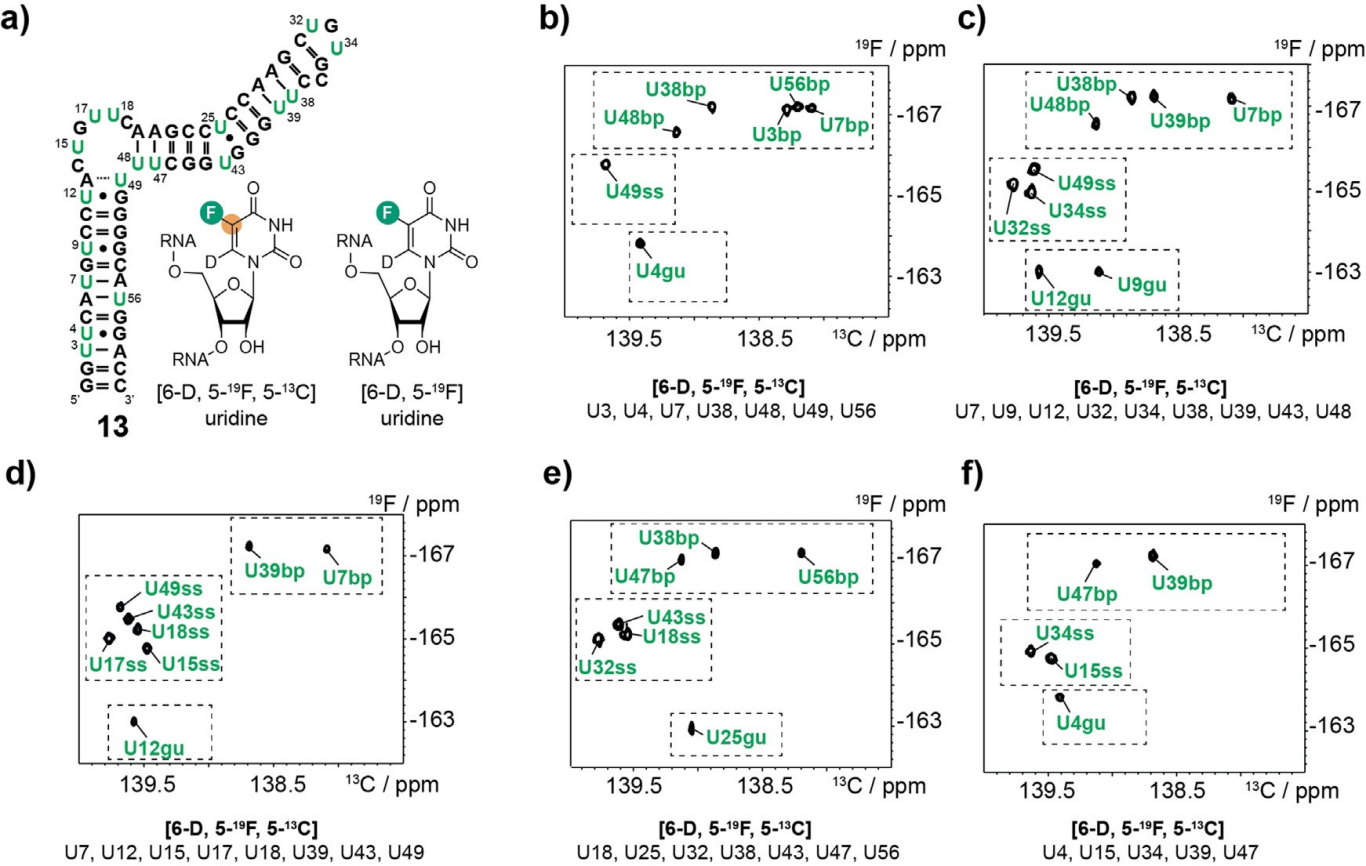
Assignment procedure for [5‐^19^F,5‐^13^C]‐uridine resonances by diluted site‐specific stable isotope labeling. a) Secondary structure representation of the hHBV *ϵ* RNA with uridines highlighted in green. The [6‐D, 5‐^19^F,5‐^13^C]‐ and [6‐D, 5‐^19^F]‐uridine labels are shown. b–f) ^19^F–^13^C‐TROSY of site‐specifically labeled RNAs. The [6‐D, 5‐^19^F,5‐^13^C] labeled Us are given, all other Us were [6‐D, 5‐^19^F] labeled. Fold specific ^19^F chemical shift regions (A–U Watson–Crick base paired bp, single stranded ss, and G–U wobble base pairs gu) are marked with dashed boxes.

We then turned to the development of an efficient resonance assignment procedure for [^19^F, ^13^C]‐labeled RNAs capitalizing on solid phase RNA synthesis to freely chose the labeling positions. In the hHBV *ϵ* RNA eighteen resonances need to be assigned. The number of sequences determines the number of assignable resonances according to the equation 2^*n*^−1 (*n*=number of sequences), that is, with four sequences 15 resonances, with five sequences 31 resonances can be assigned. The sequence design for RNA **13** is described (Supporting Information, Table S2). The five RNAs were labeled with 25 % diluted [6‐D, 5‐^19^F, 5‐^13^C]‐Us at the respective positions, whereas all other uridine residues were replaced by [6‐D, 5‐^19^F]‐Us (Figure 3 a). This 25 % SI dilution makes the approach more economic by reducing the amount of the ^13^C/^19^F‐amidite. The resonances were assigned by running TROSYs with an acquisition time of ca. 10 h per 0.5 mm RNA sample (Figure 3 b–f). The 18 [6‐D, 5‐^19^F, 5‐^13^C]‐uridine correlations could be assigned within only 50 h spectrometer time. It turned out to be crucial to conduct the resonance assignment procedure in an all [5‐^19^F]‐U‐labeled environment as neighboring Us influence the chemical shift of each other as exemplified for U3 and U4 (Figure S3). We observed the fold dependent ^19^F chemical shifts for all Us and the so far not reported shift signature for G–U wobble base pairs at −163 ppm could be confirmed for all four G–U base pairs. Based on the ^19^F shift of U49 the A13–U49 base pair is weak and forms only transiently leaving U49 mainly single stranded. An overlay of all spectra with all assignments can be found in the supporting information (Figure S3).

### [5‐^19^F, 5‐^13^C]‐Cytidine Labeling to Monitor Ligand Binding in the SAM VI Riboswitch RNA

**Figure 4:**
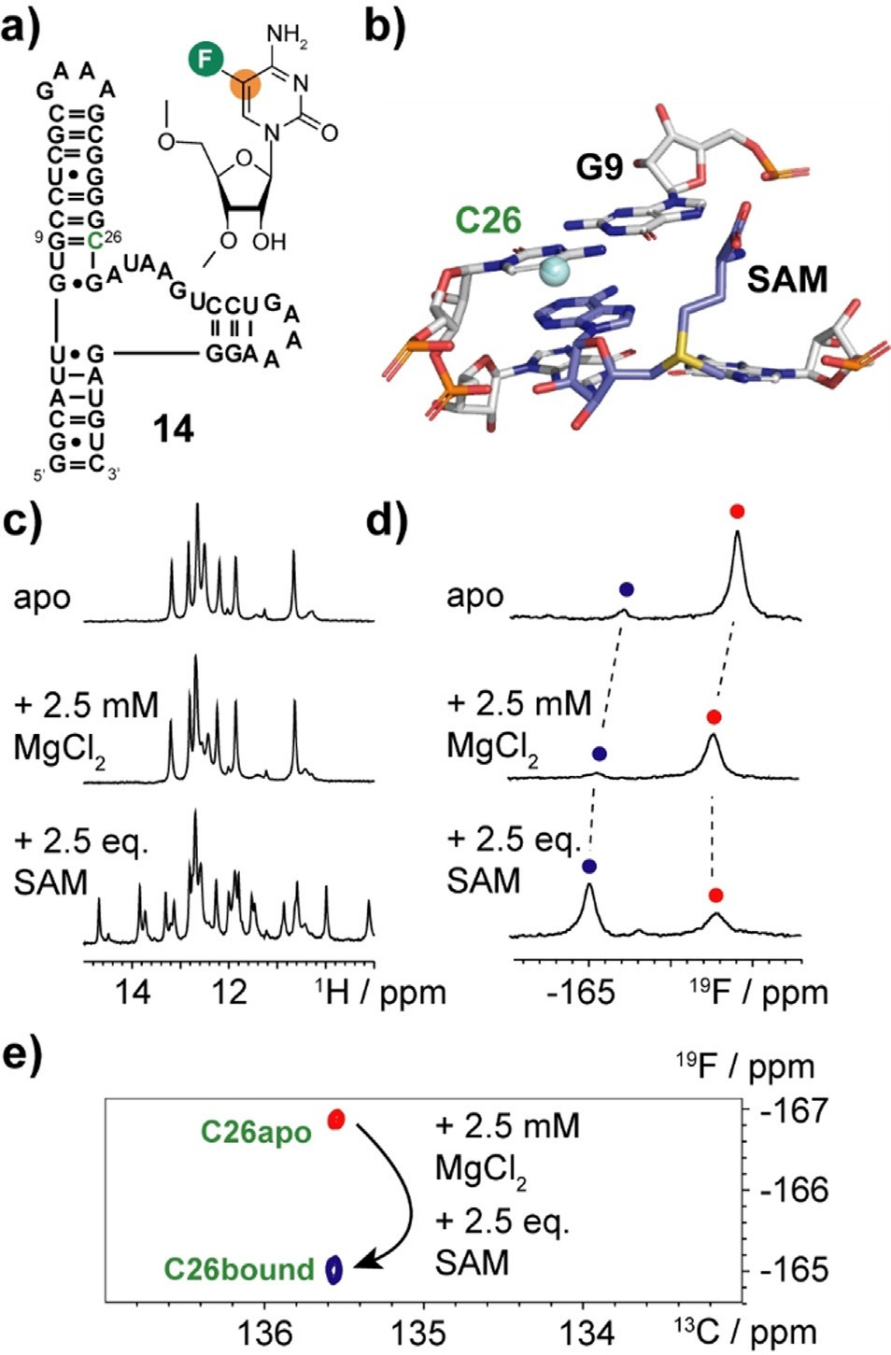
^19^F–^13^C‐TROSY to monitor ligand binding in riboswitch aptamers. a) Secondary structure representation of the SAM VI aptamer **14**. b) Part of the binding pocket from high resolution X‐ray structure. The G9–C26 base pair is shown with SAM bound and the 5‐F‐C26 label is highlighted as a sphere. c) Imino proton spectra in the apo form (upper trace), after magnesium chloride addition (middle trace) and after SAM ligand addition (lower trace). d) Same as in (c) but monitored via ^13^C‐decoupled ^19^F NMR. The apo state ^19^F resonances are highlighted with red dots, the bound‐like and bound ^19^F resonances are highlighted with a blue dot. e) Overlay of ^19^F–^13^C‐TROSYs used for the detection of ligand binding to aptamer **14**.

In the next step we exemplified the suitability of [5‐^19^F, 5‐^13^C]‐cytidine labeling to address structural and dynamic features of RNA. We picked the SAM VI riboswitch as the target and labeled the single cytidine C26 with ^19^F/^13^C (Figure 4 a). The SAM VI aptamer was discovered by Breaker and co‐workers and the 3D structure was recently determined.^10^ We picked C26 as a candidate to monitor the ligand binding event (Figure 4 b). The SAM binding pocket is composed of a Watson–Crick base pair G9–C26, one non‐canonical (*trans* Watson–Crick) G7–G27 base pair and four stacked A28, A30, A31and G32 residues and by a U8 that recognizes the SAM‐adenine base. First, the ligand binding was monitored by observing the imino proton region of ^1^H‐NMR spectra. The binding of SAM was nicely reflected, and several additional canonical and non‐canonical base pairing interactions were formed upon the complex formation (Figure 4 c). We used ^13^C‐decoupled ^19^F‐NMR to follow the ligand binding process (Figure 4 d). The C26 label not only reflected the SAM binding event but also gave insights into the binding mechanism. In the apo state we observed beside a major state (95 %) signal at −166.8 ppm another low intensity signal at −165.4 ppm with a ca. 5 % population. Based on the chemical shift signature this state originates from a ligand bound like conformational state with a closed G9–C26 base pair. The addition of magnesium(II) ions leads to minor shifts of both resonances and line broadening, but leaves the population ratio unchanged. The ligand addition leads to a novel major resonance (75 %) at −165.0 ppm, from C26 in the ligand bound state. We still observe the peak (−166.6 ppm) of the free state. This residual fraction of unbound RNA persists even upon the addition of more ligand (5 equiv.) and magnesium ions (5 mm concentration, data not shown). This could be either an effect of the fluorine modification, but given the minimally invasive nature, we think that this could represent a functional feature of the riboswitch. In‐line with NMR and fluorescence spectroscopy results on riboswitches conformational dynamics is a key feature in the ligand recognition process.^11^ Our results point toward a conformational selection mechanism for the SAM VI riboswitch, in which the 5 % ligand binding competent state of the free RNA acts as the active species for the SAM ligand docking. The co‐existence of various folding states in the presence or absence of ligand is in accordance with a regulation mechanism based on shifting equilibrium populations by external cues, such as magnesium(II) ions or the ligand, but also other factors such as pH or temperature. We recently observed a similar mechanism in the regulation of the catalytic process of the group II intron.^12^

### Combining [5‐^19^F, 5‐^13^C]‐Cytidine and ‐Uridine Labeling in the Precursor MicroRNA 21

**Figure 5:**
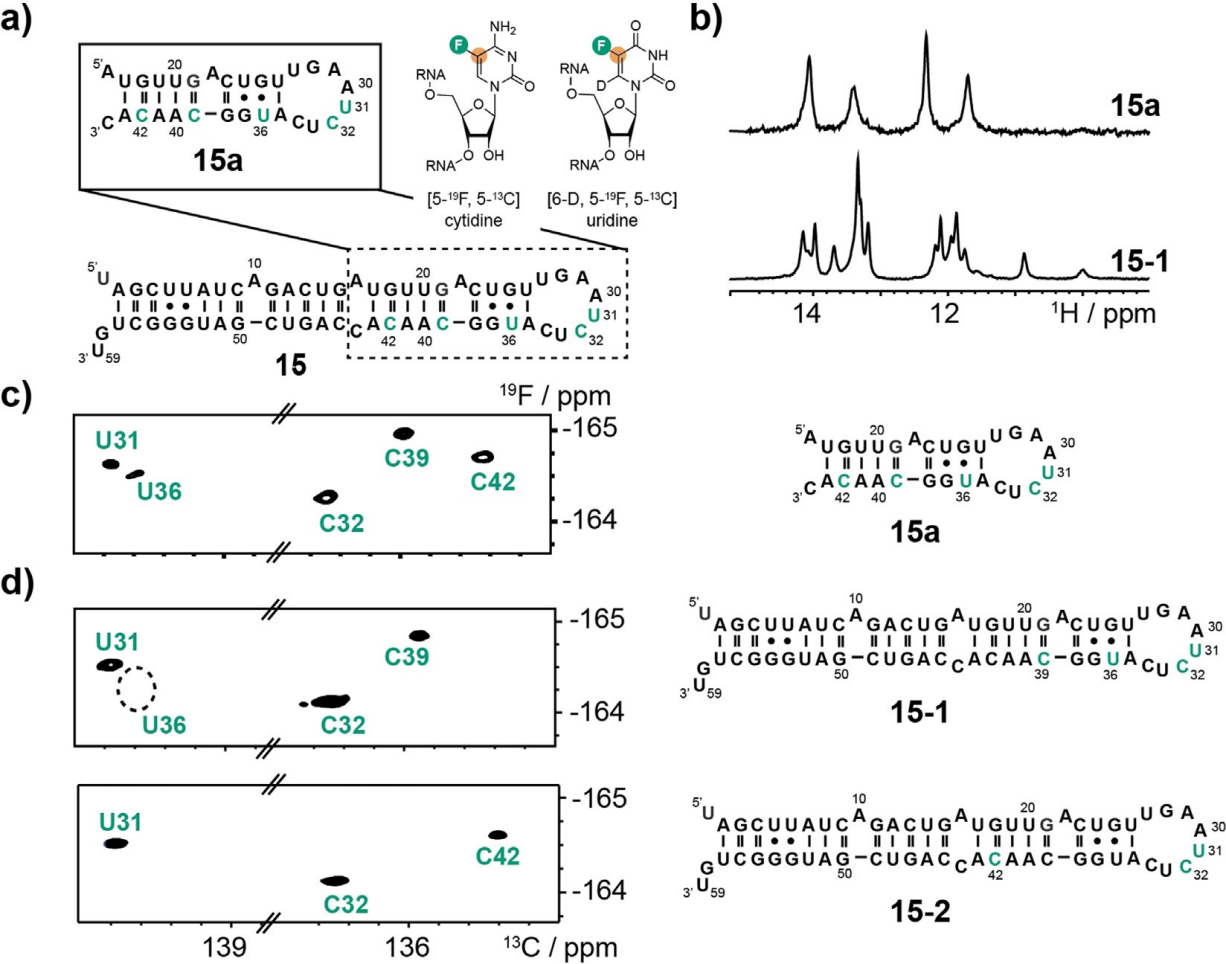
^19^F–^13^C‐cytidine and ‐uridine labeling of pre‐miRNA 21. a) Secondary structure representation of pre‐miRNA 21 **15** and the apical stem loop motif **15 a** with labeled nucleotides shown in green. The [6‐D, 5‐^19^F, 5‐^13^C]‐uridine and [5‐^19^F, 5‐^13^C]‐cytidine labels are shown as insets. b) Imino proton spectra of the 29 nt RNA **15 a** and 59 nt RNA **15** at 35 °C. c) ^19^F–^13^C‐TROSY experiment of site‐specifically labeled 29 nt RNAs **15 a** at 35 °C. The labeled residues are highlighted in green in the 2ndary structure. d) ^19^F–^13^C‐TROSYs of site‐specifically labeled 59 nt RNAs **15‐1** and **15‐2** at 35 °C. The labeled residues are highlighted in green in the secondary structure. The resonance of U36 in **15‐1** is broadened beyond detection.

As the final example the larger non‐coding pre‐miRNA 21 **15** was chosen (Figure 5 a). MicroRNAs (miRNAs) are small non‐coding RNAs that are involved in gene regulation. First, primary miRNAs are processed by the microprocessor complex in the nucleus to give precursor miRNAs (pre‐miRNAs), which are exported to the cytoplasm and cleaved by the DICER complex to give short miRNA duplexes. These duplexes are taken up by the Ago proteins and the miRNA strand is selected and the passenger strand is discarded. The Ago‐miRNA complex is the crucial component in the miRNA‐mediated RNA silencing machinery, which knocks down gene expression by targeting the mRNA. Mismatch and wobble base pairs in the upper stem of primary miRNAs were identified as crucial factors for efficient and accurate processing by the microprocessor.^13^ This is corroborated by a study of Zhang and co‐workers, in which a pH‐dependent excited state in the apical stem loop of pre‐miRNA 21 was identified.^14^ We picked the non‐coding pre‐miRNA 21 **15** as a promising target for our labeling approach in view of an application of the ^19^F/^13^C‐pyrimidines in in vivo NMR to follow the fate of pre‐miRNAs in cells. We also tested the combined U/C‐^19^F/^13^C‐labeling. The modular composition of the pre‐miRNA 21 was confirmed by an almost identical chemical shift pattern of the apical stem loop with [5‐^19^F, 5‐^13^C]‐C32, C39 and C42 labels and [6‐D, 5‐^19^F, 5‐^13^C]‐U31 and U36 labels compared to the full‐length 59 nt RNA (Figure 5 c and d). We recapitulated the conformational dynamics found by Zhang and co‐workers illustrated by U36, whose ^19^F/^13^C‐resonance was exchange broadened beyond detection at 25 °C, and was only observable at 35 °C in the 29 nt RNA **15 a** (Figure S4). The U36 resonance could not be detected in the larger pre‐miRNA **15‐1** even at 35 °C (Figure 5 d). The data is in‐line with the pH dependent switching of base pairing states by adenosine 22 protonation.^14^ We further acquired preliminary ^19^F relaxation dispersion data that allows the quantification and characterization of the alternative base pairing state (data not shown). As previously outlined, the deuteration at position 6 in the [5‐^19^F, 5‐^13^C]‐C labels was not feasible due to back exchange of the deuterium to a proton under alkaline conditions. As a consequence, the [5‐^19^F, 5‐^13^C]‐C labels experience scalar coupling interactions between the F5/C5 and the H6 leading to broadened resonances compared to the corresponding position 6 deuterated U. This is illustrated for U31 and C32 in RNA **15‐1** (Figure S5). We, thus, recommend for larger RNAs to mainly use the [6‐D, 5‐^19^F, 5‐^13^C]‐U labeling protocol, especially if proton decoupling is not feasible with the available NMR hardware as in our case.

## Conclusion

We present an access to a [5‐^19^F, 5‐^13^C]‐cytidine and a [5‐^19^F, 5‐^13^C]‐uridine phosphoramidite suitable to modify RNAs with ^19^F/^13^C‐spin topologies. A regioselective fluorination of [5‐^13^C]‐uracil using Selectfluor is the key step to give [5‐^19^F, 5‐^13^C]‐uracil. The nucleobase is fused to a sugar unit under silyl‐Hilbert‐Johnson conditions. Optionally, the proton H6 can be deuterated at this stage to minimize the scalar coupling network of the fluorine and to give sharper lines with a higher signal to noise ratio. The deuteration at position 6 can only be retained in the uridine building block, whereas for the cytidine we observed a re‐protonation at position 6 during the U to C transformation. We further anticipate that this re‐protonation would also occur during the alkaline deprotection step after the RNA solid phase synthesis leading to a mixture of C6‐deuterated and ‐protonated [5‐^19^F, 5‐^13^C]‐cytidines. Thus, we decided to only synthesize the [5‐^19^F, 5‐^13^C]‐cytidine phosphoramdite **11**. Standard transformations allowed the synthesis of the phosphoramidites **7** and **11**. We found it crucial to use pyridine instead of triethylamine as eluent additive for silica column chromatography to obtain reproducible yields. This is attributed to the higher acidity of the H3 imino proton in [5‐^19^F]‐modified U, which can react with triethylamine to give the ammonium salt. Once the RNA phosphoramidites **7** and **11** were available RNAs with sizes between 30 and 60 nts were synthesized. First, the 30 nt HIV TAR‐2 with six uridines and the 61 nt hHBV *ϵ* RNA with eighteen uridines were synthesized and six and five [5‐^19^F, 5‐^13^C]‐U labels were incorporated in each RNA, respectively. We obtained high quality ^19^F–^13^C TROSY spectra within a short time on a Prodigy TCI probe with the proton coil tuned to the ^19^F resonance frequency for 0.5 mm RNA samples. The additional ^13^C dimension allowed to resolve the resonance overlap, which was earlier observed for U23, U25 and U31 in the 1D‐^19^F spectrum.2c With only 10 kD RNA **12** did not show a pronounced TROSY effect. Thus, the TROSY properties of the FC‐U labels were probed in the 61 nt hHBV *ϵ* RNA with ≈20 kD. Here, we observed a more pronounced TROSY effect manifested in the ^19^F spectrum without ^13^C decoupling. We qualitatively compared a ^13^C‐detected out‐and‐stay TROSY experiment with a ^1^H‐^13^C TROSY experiment of a [5‐D, 6‐^13^C]‐uridine labeled 61 nt hHBV *ϵ* RNA. Both stable isotope labeling schemes gave well resolved correlation peaks at 25 °C but at 0 °C, where the slowed down molecular mobility leads to a higher molecular weight like behavior, the superior spectral properties of the ^19^F/^13^C spin pair were manifested. All five [6‐D, 5‐^19^F, 5‐^13^C]‐U labels could be observed, in contrast only two of five ^1^H‐^13^C uridine resonances were found (Figure S6). We also assigned all eighteen ^19^F–^13^C uridine resonances of the 61 nt hHBV *ϵ* RNA. The resonance assignment confirmed the fold specific ^19^F chemical shifts in [5‐^19^F]‐uridines.2c The analysis of the ^19^F chemical shifts not only allows to discriminate single stranded and A–U base paired uridines, but we also found a distinct chemical shift signature for G⋅U wobble pairs.

We incorporated a single [5‐^19^F, 5‐^13^C]‐C label into the SAM VI aptamer and could follow the ligand binding process and get insights into the recognition process. We observed a minor ^19^F‐resonance indicating that the binding competent state is already present in the apo RNA supporting a conformational selection mechanism. In the final RNA target—the 59 nt pre‐miRNA 21—[6‐D, 5‐^19^F, 5‐^13^C]‐U and [5‐^19^F, 5‐^13^C]‐C labeling was combined. Both labels gave satisfactory results but in larger RNAs the deuteration of position 6 in the U‐building block gave sharper linewidths and a better signal to noise ratio, as in our NMR hardware setup proton decoupling cannot be accomplished.

To sum up, the novel [6‐D, 5‐^19^F, 5‐^13^C]‐U and [5‐^19^F, 5‐^13^C]‐C labels can be used to quickly screen folding states, ligand binding events and structural and dynamics features of RNA not only under in vitro but also under in vivo conditions facilitated by fluorine's bioorthogonality. Altogether, we anticipate that the stable isotope labeling Scheme introduced in this work is highly attractive for in vivo NMR applications of RNAs in combination with dynamic nuclear polarization to boost sensitivity.^15^

## Conflict of interest

The authors declare no conflict of interest.

## Supporting information

As a service to our authors and readers, this journal provides supporting information supplied by the authors. Such materials are peer reviewed and may be re‐organized for online delivery, but are not copy‐edited or typeset. Technical support issues arising from supporting information (other than missing files) should be addressed to the authors.

Supplementary
