# Supplementary material for: Aromatic 19F–13C TROSY—[19F, 13C]‐Pyrimidine Labeling for NMR Spectroscopy of RNA
Source: Angew Chem Int Ed Engl. 2020 Jul 29;59(39):17062–9. doi: 10.1002/anie.202006577 (PMC7540360; doi:10.1002/anie.202006577)
Supplement: Supplementary file 1 — Supplementary [file ANIE-59-17062-s001.pdf]

Angewandte Chemie
Eine Zeitschrift der Gesellschaft Deutscher Chemiker

Supporting Information

Aromatic ^{19}F–^{13}C TROSY—[^{19}F, ^{13}C]-Pyrimidine Labeling for NMR Spectroscopy of RNA

*Felix Nußbaumer[+], Raphael Plangger[+], Manuel Roeck, and Christoph Kreutz**

anie_202006577_sm_miscellaneous_information.pdf

Synthesis of [5-^{19}F, 5-^{13}C] uridine phosphoramidite 7

[5-^{19}F, 5-^{13}C]-uracil (2). A suspension of [5-^{13}C]-uracil **1** (1.0 eq., 1.0 g, 8.92 mmol) together with Selectfluor^{TM} (F-TEDA, 1.0 eq., 3.16 g, 8.92 mmol) in 30 mL of water was stirred overnight at 90°C under an argon atmosphere. Then, to the still warm mixture, was added sodium tetraphenylborate (2.20 eq., 6.72 g, 19.63 mmol) as a solid at once and stirring was continued for 5 minutes. The mixture was stored on ice for 30 minutes and the solid material was filtered off over a pad of celite and washed with 3 x 10 mL water. The filtrate was evaporated to a solid residue and dried in high vacuum at 90°C overnight. The solid material was finely pulverized. Sublimation of the crude product at 210°C and 0.01 mbar gave **4** with 10% (5-^{13}C)-uracil as impurity. Yield: 0.55 g (4.26 mmol, 47%); ^{1}H-NMR (400 MHz, DMSO-d$_6$, 25°C): δ 11.50 (bs, 1H, NH); 10.71 (bs, 1H, NH); 7.75 (m, 1H, $^3J_{FH}$ = 4.20 Hz, $^2J_{CH}$ = 6.15 Hz, C(6)) ppm; ^{13}C-NMR (100 MHz, DMSO-d$_6$, 25°C): δ 158.0 (dd, $^1J_{CC}$ = 80.6 Hz, $^2J_{CF}$ = 25.7 Hz, C(4)); 150.2 (C(2)); 140.7 (d, $^1J_{CF}$ = 226.9 Hz, ^{13}C(5)F); 126.4 (dd, $^1J_{CC}$ = 89.3 Hz, $^2J_{CF}$ = 31.3 Hz, C(6)) ppm; ^{19}F-NMR (375 MHz, DMSO-d$_6$, 25°C): δ -171.5 (dd, $^1J_{CF}$ = 226.8 Hz, $^3J_{FH}$ = 4.20 Hz, ^{13}C(5)F) ppm.

5',3',2'-Tri-*O*-benzoyl-[5-^{19}F, 5-^{13}C]-uridine (3). Compound **2** (1.0 eq., 1.60 g, 12.30 mmol) together with 1-*O*-Acetyl-2,3,5-tri-*O*-benzoyl-*β-D*-ribofuranose (ATBR, 1.0 eq., 6.21 g, 12.30 mmol) was co-evaporated two times with anhydrous acetonitrile. To the residue suspended in 50 mL of dry acetonitrile was added *N*,*O*-bis(trimethylsilyl)acetamide (BSA, 3.0 eq., 7.51 g, 36.90 mmol) under an argon atmosphere. The mixture was heated to 60°C with stirring for 30 minutes. To the solution was added trimethylsilyl trifluoromethanesulfonate (TMSOTf, 3.5 eq., 9.57 g, 43.05 mmol) and stirring was continued for 30 minutes at 60°C. Then, thin layer chromatography showed complete conversion. The mixture was evaporated to give an oily residue, dissolved in chloroform and washed twice with saturated sodium bicarbonate solution. The organic phase was dried over anhydrous sodium sulfate, filtered and evaporated to dryness. The crude product was applied to a silica gel column with methylene chloride and eluted using a gradient from 0.5 to 5% methanol in methylene chloride to give **3** as a yellow solid. The product was dried in high vacuum. Yield: 4.46 g (7.70 mmol, 64%); TLC: CH_2Cl_2/MeOH = 9.5/0.5) R_f = 0.5; ^{1}H-NMR (400 MHz, DMSO-d$_6$, 25°C): δ 12.02 (s, 1H, NH); 8.21 (dd, 1H, $^3J_{FH}$ = 6.85 Hz, $^2J_{CH}$ = 5.10 Hz, C(6)H); 8.00 (m, 2H, C(ar)H); 7.88 (m, 4H, C(ar)H); 7.68 – 7.62 (m, 3H, C(ar)H); 7.52 – 7.42 (m, 6H, C(ar)H); 6.17 (d, 1H, C(1')H); 5.91 (m, 2H, C(2')H, C(3')H); 4.74 (m, 1H, C(4')H); 4.69 (m, 2H, C(5')H$_2$) ppm; ^{13}C-NMR (100 MHz, DMSO-d$_6$, 25°C): δ 165.5 (C q); 164.6 (C q); 149.0 (C q); 140.3 (d, $^1J_{CF}$ = 231.5 Hz, ^{13}C(5)F); 133.9 , 133.8, 133.6, 129.4, 129.3, 128.8, 128.5, 128.4 (C(ar)H; 126.0 (dd, $^1J_{CC}$ = 91.4 Hz, $^2J_{CF}$ = 33.6 Hz, C(6)); 88.9 (C(1')); 78.9 (C(4')); 73.1, 70.3 (C(2'), C(3')); 63.7 (C(5')) ppm; ^{19}F-NMR (375 MHz, DMSO-d$_6$, 25°C): δ -171.5 (dd, $^1J_{CF}$ = 232.2 Hz, $^3J_{FH}$ = 6.84 Hz, ^{13}C(5)F) ppm; ESI-MS (m/z): calculated for $^{13}CC_{30}H_{23}FN_2O_9$ [M + Na]$^+$: 598.1313, found: 598.1292 [M+H]$^+$.

[5-^{19}F, 5-^{13}C]-uridine (4). Compound **3** (4.46 g, 7.70 mmol) was treated with of 100 mL methylamine solution (33 wt% in absolute ethanol) and stirred at room temperature overnight. The mixture was evaporated to dryness and dried in high vacuum for 30 minutes. The residue was dissolved in a minimum of warm methanol/methylene chloride (1:1) and added dropwise to 300 mL of an ice-cooled mixture of methylene chloride/diethyl ether/hexane (1:1:1) with vigorous stirring. The product

precipitates immediately and was stored at 4°C overnight. The product was filtered off, washed with methylene chloride and dried in high vacuum to give **4** as a yellow solid. Yield: 1.80 g (6.87 mmol, 88%); TLC: CH_2Cl_2/MeOH = 8/2) R_f = 0.2; ^{1}H-NMR (400 MHz, DMSO-d_6, 25°C): δ 8.18 (dd, 1H, $^3J_{FH}$ = 7.25 Hz, $^2J_{CH}$ = 4.68 Hz, C(6)H); 5.74 (d, 1H, C(1')H); 4.74 (d, 1H, C(1')H); 3.99 (m, 2H, C(2')H, C(3')H); 3.83 (m, 1H, C(4')H); 3.66, 3.57 (m, 2H, C(5')H_2) ppm; ^{13}C-NMR (100 MHz, DMSO-d_6, 25°C): δ 158.6 (dd, $^1J_{CC}$ = 80.6 Hz, $^2J_{CF}$ = 25.7 Hz, C(4)); 150.5 (C(2)); 140.3 (d, $^1J_{CF}$ = 231.6 Hz, ^{13}C(5)F); 124.3 (dd, $^1J_{CC}$ = 77.6 Hz, $^2J_{CF}$ = 24.2 Hz, C(6)); 88.3 (C(1')); 84.6 (C(4')); 73.8 (C(2'), 69.5 C(3')); 60.5 (C(5')) ppm; ^{19}F-NMR (375 MHz, DMSO-d_6, 25°C): δ -167.1 (dd, $^1J_{CF}$ = 232.1 Hz, $^3J_{FH}$ = 7.30 Hz, ^{13}C(5)F) ppm; ESI-MS (m/z): calculated for $^{13}CC_8H_{11}FN_2O_6$ [M + Na]$^+$: 286.0527, found: 286.0516 [M+H]$^+$.

3',5'-*O*-bis(*tert.*-butylsilyl)-2'-*O*-(*tert.*-butyldimethylsilyl)-[5-^{19}F,5-^{13}C]-uridine (5). Compound **4** (1.0 eq., 1.80 g, 6.87 mmol) was dried overnight in high vacuum at 80 °C. The dried compound was suspended in 20 mL of dry DMF and di-*tert*.-butylsilyl bis(trifluoromethanesulfonate) (1.1 eq., 3.33 g, 7.55 mmol) was added dropwise with stirring at 0°C under an argon atmosphere. After 10 minutes thin layer chromatography showed complete conversion to the intermediate and the suspension turned into solution. Imidazole (5.0 eq., 2.34 g, 34.33 mmol) was added at once at 0°C and the mixture was allowed to warm to room temperature. Then, *tert*.-butyl dimethylsilyl chloride (1.2 eq., 1.24 g, 8.24 mmol) was added at once and the mixture was stirred for 2 h at 60°C until thin layer chromatography showed complete conversion. DMF was distilled off in high vacuum, the oily residue dissolved in chloroform and washed twice with brine. The organic layer was dried over anhydrous sodium sulfate, filtered, and evaporated to dryness. The crude product was applied to a silica gel column with ethyl acetate/*n*-hexane 2:8 and eluted using a gradient from 2:8 to 4:6 ethyl acetate/n-hexane to give **5** as a colorless solid. The product was dried in high vacuum. Yield: 2.52 g (4.80 mmol, 70%); TLC: ethyl acetate/n-hexane = 3/7) R_f = 0.5; ^{1}H-NMR (400 MHz, DMSO-d_6, 25°C): δ 11.93 (bs, 1H, NH); 7.92 (dd, 1H, $^3J_{FH}$ = 5.11 Hz, $^2J_{CH}$ = 7.63 Hz, C(6)H); 5.64 (d, 1H, C(1')H); 4.39 (m, 1H, C(2')H); 4.36 (m, 1H, C(5')H); 4.11 – 4.05 (m, 2H, C(3')H, C(5')H); 3.89 (m, 1H, C(3')H); 1.03, 0.98 (2 x s, 18H, Si(tBu)$_2$, DTBS), 0.88; (s, 9H, Si-tBu, TBDMS) 0.12, 0.08 (2 x s, 6H, Si-(CH_3)$_2$, TBDMS) ppm; ^{13}C-NMR (100 MHz, DMSO-d_6, 25°C): δ 148.69 (C q); 140.2 (d, $^1J_{CF}$ = 231.4 Hz, ^{13}C(5)F); 124.97 (C(6)); 93.02 (C(1')); 74.95 (C4')), 74.25 (C(2')); 73.92 (C(3')); 66.60 (C(5'); 27.34, 26.78 (Si(tBu)$_2$, CH_3, DTBS) 22.09 (C q, tBu); 19.94 (C, tBu); 18.00 (Cq, tBu); -4.51, -5.21 (2 x Si(CH_3)$_2$) ppm; ^{19}F-NMR (375 MHz, DMSO-d_6, 25°C): δ -167.1 (dd, $^1J_{CF}$ = 230.9 Hz, $^3J_{FH}$ = 5.29 Hz, ^{13}C(5)F) ppm; ESI-MS (m/z): calculated for $^{13}CC_{22}H_{41}FN_2O_6Si_2$ [M + Na]$^+$: 540.2413, found: 540.2411 [M+H]$^+$.

2'-*O*-(*tert.*-butyldimethylsilyl)-5'-*O*-(4,4'-dimethoxytrityl)-[5-^{19}F,5-^{13}C]-uridine (6). Compound **5** (1.29 g, 2.50 mmol) was dissolved in 20 mL of dry methylene chloride. To this solution was added dropwise at 0°C under stirring a premixed solution of 225 µL HF-Pyridine (70 % hydrogen fluoride basis, 30 % pyridine basis) and 1.30 mL pyridine. After stirring 2h at 0°C thin layer chromatography showed complete conversion (ethyl acetate/n-hexane = 7/3; R_f = 0.2). The mixture was allowed to warm to room temperature, and was diluted with chloroform and washed once with saturated sodium bicarbonate solution. The organic layer was dried over anhydrous sodium sulfate, filtered and evaporated to dryness. The crude product was dried in high vacuum and used without further purification for the next

step. The crude product (1.0 eq., 2.50 mmol) together with one spatula tip of 4-(dimethylamino)pyridine was co-evaporated twice with anhydrous pyridine and then dissolved in 25 mL of dry pyridine. Then 4,4'-dimethoxytrityl chloride (1.1 eq., 932 mg, 2.75 mmol) was added in three portions within 1h and the mixture was stirred for 3h at room temperature. The mixture was quenched with 1 mL of methanol, evaporated to an oily residue and two times co-evaporated with toluene. The residue was dissolved in chloroform and washed two times with 5% citric acid and once with saturated sodium bicarbonate solution. The organic layer was dried over anhydrous sodium sulfate, filtered and evaporated to dryness. The crude product was applied to a silica gel column with methylene chloride and eluted using a gradient from 0 to 3% methanol in methylene chloride to give **6** as a yellowish foam. The product was dried in high vacuum. Yield: 800 mg (1.18 mmol, 47% over two steps); TLC: CH_2Cl_2/MeOH = 9/1) R_f = 0.6; ^{1}H-NMR (400 MHz, $CDCl_3$, 25°C): δ 9.08 (bs, 1H, NH); 7.81 ($^3J_{FH}$ = 4.80 Hz, $^2J_{CH}$ = 5.91 Hz, C(6)H); 7.25 (m, 2H, C(ar)H); 7.16 – 7.09 (m, 5H, C(ar)H); 6.71 – 6.68 (m, 4H, C(ar)H); 5.80 (d, 1H, C(1')H); 4.27 (t, 1H, C(2')H); 4.20 (q, 1H, C(3')H); 4.01 (m, 1H, C(4')H); 3.77 (m, 2H, 3.64 (s, 6H, 2 x O-CH_3); 3.43 (m, 2H, 2 x iPr); 3.33 (m, 2H, C(5')H_2); 1.06 (m, 12H, 2 x iPr); 0.79 (s, 9H, Si-tBu); 0,02 (s, 6H, Si(CH_3)$_2$) ppm; ^{13}C-NMR (100 MHz, $CDCl_3$, 25°C): δ 158.7 (C q); 158.7 (C q); 144.2 (C q); 140.6 (d, $^1J_{CF}$ = 239.1 Hz, ^{13}C(5)F); 135.2 (C q); 134.9 (C q); 124.5 – 120.8 (C(ar)H); 118.0 (C(6)H); 82.3 (C(1')); 77.8 (C(4)'); 70.2 (C(2')); 65.3 (C(3')); 56.9 (C(5')); 19.1 (C, tBu), -4,87 (2 x Si(CH_3)$_2$) ppm; ^{19}F-NMR (375 MHz, $CDCl_3$, 25°C): δ -163.7 (dd, $^1J_{CF}$ = 239.1 Hz, $^3J_{FH}$ = 5.11 Hz, ^{13}C(5)F) ppm; ESI-MS (m/z): calculated for $^{13}CC_{35}H_{43}FN_2O_8Si$ [M + Na]$^+$: 702.2698, found: 702.2708 [M+H]$^+$.

2'-*O*-(*tert.*-butyldimethylsilyl)-5'-*O*-(4,4'-dimethoxytrityl)-[5-^{19}F,5-^{13}C]-uridine 3'-[(2-cyanoethyl)-(*N,N*-diisopropyl)]phosphoramidite (7). Compound **6** (1.0 eq., 800 mg, 1.18 mmol) was dissolved in 20 mL of dry acetonitrile. To this solution was added 5-(benzylthio)-1*H*-tetrazole (BTT, 2.0 eq., 452 mg, 2.36 mmol), activated 3 Å molecular sieve (2.0 g) and the mixture was stirred 2h under an argon atmosphere. To this was added 2-cyanoethyl *N,N,N',N'*-tetraisopropylphosphorodiamidite (2.0 eq., 709 mg, 2.36 mmol) and stirring was continued overnight at room temperature. The mixture was diluted with chloroform and washed once with half saturated sodium bicarbonate solution. The organic layer was dried over anhydrous sodium sulfate, filtered and evaporated to dryness. The crude product was applied to a silica gel column with ethyl acetate/n-hexane 1:9 (+ 1 % pyridine) and eluted with 1:1 ethyl acetate/n-hexane (+ 1 % pyridine) to give **7** as a colorless foam. The product was dried in high vacuum. Yield: 924 mg (1.05 mmol, 89%); TLC: ethyl acetate/n-hexane = 1/1 + 1 % pyridine) R_f = 0.7; ^{1}H-NMR (400 MHz, $CDCl_3$, 25°C): δ 7.85 (m, 1H, C(6)H); 7.44 – 7.21 (m, 9H, C(ar)H)); 6.84 (m, 3H, C(ar)H); 5.98 (m, 1H, C(1')H); 5,78 ((m, 2H, 2 x NC*H*-(CH_3)$_2$); 3.72, 2.49 ((m, 2H, PO-C*H*$_2$); 3.49, 2.37 (2H; CN-C*H*$_2$) 4.31 (m, 1H, C(2')H); 4.21, 4.14 (m, 1H, C(3')H); 3.97 (m, 1H, C(4')H); 3.45, 3.40 (m, 2H, C(5')H); 1.17 (m, 12H, 2 x NCH-(C*H*$_3$)$_2$); 0.92 (m, 9H, Si-tBu); 0.14 (m, 6H, 2 x Si(C*H*$_3$)$_2$) ppm; ^{13}C-NMR (100 MHz, $CDCl_3$, 25°C): δ 130.7 (d, $^1J_{CF}$ = 238.1 Hz, ^{13}C(5)F) ppm; ^{31}P-NMR (162 MHz, CDCl3, 25°C): δ 150.3; 149.3 ppm; ^{19}F-NMR (375 MHz, $CDCl_3$, 25°C): δ -164.6 (dd, $^1J_{CF}$ = 238.9 Hz, $^3J_{FH}$ = 6.2 Hz, ^{13}C(5)F) ppm; ESI-MS (m/z): calculated for $^{13}CC_{44}H_{60}FN_4O_9PSi$ [M + Na]$^+$: 902.3777, found: 902.3734 [M+H]$^+$.

Synthesis of [5-^{19}F, 5-^{13}C] cytidine phosphoramidite 11

3',5'-*O*-bis(*tert.*-butylsilyl)-2'-*O*-(*tert.*-butyldimethylsilyl)-[5-^{19}F,5-^{13}C]-cytidine (8). Compound **5** (1.0 eq., 1.3 g, 2.51 mmol) was dissolved in 35 mL of dry acetonitrile. Triethylamine (10 eq., 2.54 g, 3.5 mL) and a catalytic amount of *N*,*N*-dimethylaminopyridine (DMAP, 0.2 eq., 61.4 mg, 502 µmol) were added at 0°C and the reaction mixture was stirred under argon for 15 minutes. Then, 2,4,6-triisopropylbenzenesulfonyl chloride (2 eq., 1.52 g, 5.02 mmol) was added in 3 portions over 1h. The reaction was allowed to proceed for 2h at room temperature and the progress was monitored by TLC (*n*-hexanes/ethyl acetate 1/1, R_f = 0.9). After 2h at room temperature, 40 mL acetonitrile and 30 mL of 18% aqueous ammonia solution were added and stirring was continued overnight. The reaction mixture was then evaporated and the residue dissolved in methylene chloride. The organic phase was washed with saturated sodium bicarbonate solution and then dried over anhydrous sodium sulfate, filtered and evaporated to dryness. The crude product was applied to a silica gel column with methylene chloride/methanol from 99/1 90/10 to give **8** as a colorless foam. The product was dried in high vacuum. Yield: 1.05 g (1.99 mmol, 79%); TLC: methylene chloride/methanol = 9/1 R_f = 0.6; ^{1}H-NMR (400 MHz, DMSO-d_6, 25 °C): δ 7.86, 7.60 (d, 2H, C(4)NH2); 7.83-7.80 (dxd, 1H, $^2J_{CH}$ = 4.34 Hz, $^3J_{FH}$ = 6.94 Hz, C(6)H); 5.64 (s, 1H, C(1')H); 4.37-4.29 (m, 2H, C(2'), C(3')); 4.17-3.99 (m, 3H, C(4')H, C(5')H, C(5")H); 1.04, 0.99 (2s, 18H, Si(tBu)2, bis-TBS); 0.87 (s, 9H, Si-tBu, TBDMS); 0.12, 0.078 (2s, 6H, Si-(CH3)2, TBDMS) ppm; ^{13}C-NMR (100 MHz, DMSO-d6, 25 °C): δ 137.4 (d, $^1J_{CF}$ = 242.2 Hz ^{13}C(5)F) ppm; ^{19}F-NMR (375 MHz, DMSO-d_6, 25 °C): δ -166.79 (dxd, $^1J_{CF}$ = 242.2 Hz, $^3J_{FH}$ = 6.94 Hz, 13C(5)F) ppm.

4-*N*-acetyl-3',5'-*O*-bis(*tert.*-butylsilyl)-2'-*O*-(*tert.*-butyldimethylsilyl)-[5-^{19}F,5-^{13}C]-cytidine (9). Compound **8** (1.0 eq., 1.68 g, 3.25 mmol) was dissolved in 15 mL of dry *N*,*N*-dimethylformamide (DMF) and acetic anhydride (1.5 eq., 499 mg, 461 µL) was added under argon. The reaction was stirred for 24h at room temperature and then quenched by the addition of 2 mL methanol. The reaction mixture was evaporated under high vacuum and at 60°C. The residue was taken up in methylene chloride and the organic phase was washed with saturated bicarbonate solution, followed by washing with brine (three times). The organic phase was dried over anhydrous sodium sulfate, filtered and evaporated to dryness. The crude product was applied to a silica gel column with *n*-hexanes/ethyl acetate from 9/1 4/6 to give **9** as a colorless foam. The product was dried in high vacuum. Yield: 1.52 g (2.72 mmol, 84%); TLC: n-hexanes/ethyl acetate = 1/1 R_f = 0.4; ^{1}H-NMR (400 MHz, DMSO-d_6, 25 °C): δ 10.5 (s, NH, C(4)NH); 8.04-7.99 (dxd, 1H, $^2J_{CH}$ = 4.31 Hz, $^3J_{FH}$ = 5.92 Hz, C(6)H); 5.63 (s, 1H, C(1')H); 4.41-4.34 (m, 2H, C(2'), C(3')); 4.19 (m, 1H, C(4')H) 4.07-3.94 (m, 2H, C(5')H, C(5")H); 2.24 (NAc); 1.03, 0.99 (2s, 18H, $Si(tBu)_2$, bis-TBS); 0.90 (s, 9H, Si-tBu, TBDMS); 0.16, 0.11 (2s, 6H, Si-$(CH_3)_2$, TBDMS) ppm; ^{13}C-NMR (100 MHz, DMSO-d_6, 25 °C): δ 138.2 (d, $^1J_{CF}$ = 244.8 Hz ^{13}C(5)F); 94.4 (C(1')) ppm; ^{19}F-NMR (375 MHz, DMSO-d_6, 25 °C): δ -158.3 (dxd, $^1J_{CF}$ = 244.8 Hz, $^3J_{FH}$ = 6.90 Hz, ^{13}C(5)F) ppm. ESI-MS (m/z): calculated for $^{13}CC_{24}H_{44}FN_3O_6Si_2$ [M + H]$^+$: 559.2859, found: 559.2861 [M+H]$^+$.

4-*N*-acetyl-2'-*O*-(*tert.*-butyldimethylsilyl)-5'-*O*-(4,4'-dimethoxytrityl)-[5-^{19}F,5-^{13}C]-cytidine (10). Compound **9** (1.0 eq., 1.52 g, 2.71 mmol) was dissolved in 30 mL of dry methylene chloride and cooled to 0°C. Then, 10% HF-pyridine (3.85 eq., 10.75mmol, 2 mL,) was added under argon and stirring was continued for 2.5h at 0°C. The reaction mixture was diluted with methylene chloride and the organic phase was washed with saturated bicarbonate solution, water and 5% citric acid. The organic phase

was dried over anhydrous sodium sulfate, filtered and evaporated to dryness. The crude product was dried in high vacuum for 1h. The crude product was co-evaporated three times with anhydrous pyridine and then 4,4'-dimethoxytrityl chloride (1.5 eq., 1.4 g, 4.08 mmol) was added in three portions within 1h and the mixture was stirred for 16h at room temperature. The mixture was quenched with 1 mL of methanol, evaporated to an oily residue and two times co-evaporated with toluene. The residue was dissolved in chloroform and washed two times with 5% citric acid and once with saturated sodium bicarbonate solution. The organic layer was dried over anhydrous sodium sulfate, filtered and evaporated to dryness. The crude product was applied to a silica gel column and eluted using a gradient from 30 to 60% ethyl acetate in n-hexanes to give **10** as a yellowish foam. The product was dried in high vacuum. Yield: 1.25 g (1.73 mmol, 48% over two steps); ^{1}H-NMR (400 MHz, DMSO-d_6, 25 °C): δ 10.5 (s, NH, C(4)NH); 8.21-8.14 (dxd, 1H, $^{2}J_{CH}$ = 4.03 Hz, $^{3}J_{FH}$ = 6.22 Hz, C(6)H); 7.43-7.16 (m, 10H, CH(ar)); 6.93-6.86 (m, 4H, CH(ar)); 5.61 (s, 1H, C(1')H); 5.15-5.10 (d, 1H, 3'OH); 4.20 (m, 1H, C(2')); 4.15 (m, 1H, C(3')H); 4.08 (m, 1H) 4.01 (m, 1H, C(4')); 3.74 (s, 6H, DMT(OCH_3)); 2.22 (s, 3H, NAc); 0.88 (s, 9H, Si-tBu, TBDMS); 0.12, 0.082 (2s, 6H, Si-$(CH_3)_2$, TBDMS) ppm; ^{13}C-NMR (100 MHz, DMSO-d_6, 25 °C): δ 159.1 (CH(ar)); 138.2 (d, $^{1}J_{CF}$ = 245.5 Hz ^{13}C(5)F); 129.7 (CH(ar)); 127.6 (CH(ar)); 113.3 (CH(ar)); 90.8 (C(1')); 76.1 (C(2')); 54.6 (OCH_3); 25.7 (Si-C-CH_3 TBDMS); 24.7 (HN(C=O)CH_3); 18.1 (Si-CH_3)$_3$ TBDMS); -5.1 (Si-$(CH_3)_2$) ppm; ^{19}F-NMR (375 MHz, DMSO-d6, 25 °C): δ -158.3 (dxd, $^{1}J_{CF}$ = 245.5 Hz, $^{3}J_{FH}$ = 4.03 Hz, ^{13}C(5)F) ppm; ESI-MS: calculated for $^{13}CC_{37}H_{46}FN_3O_8Si$ $[M+H]^+$: 720.3072, found 721.3081 $[M+H]^+$.

4-*N*-acetyl-2'-*O*-(*tert.*-butyldimethylsilyl)-5'-*O*-(4,4'-dimethoxytrityl)-[5-^{19}F,5-^{13}C]-cytidine 3'-[(2-cyanoethyl)-(*N,N*-diisopropyl)]phosphoramidite (11). Compound **10** (1.0 eq., 1.25 g, 1.73 mmol) was dissolved in 10 mL of dry acetonitrile. To this solution was added 5-(benzylthio)-1*H*-tetrazole (BTT, 1.2 eq., 400 mg, 2.08 mmol), activated 3 Å molecular sieve (2.0 g) and the mixture was stirred 2h under an argon atmosphere. To this was added 2-cyanoethyl *N,N,N',N'*-tetraisopropylphosphorodiamidite (2.5 eq., 1.31 g, 4.34 mmol) and stirring was continued overnight at room temperature. The mixture was diluted with chloroform and washed once with half saturated sodium bicarbonate solution. The organic layer was dried over anhydrous sodium sulfate, filtered and evaporated to dryness. The crude product was applied to a silica gel column with ethyl acetate/*n*-hexanes 1:1 (+ 1 % pyridine) and eluted with 6:4 ethyl acetate/*n*-hexanes (+ 1 % pyridine) to give **11** as a colorless foam. The product was dried in high vacuum. Yield: 470 mg (0.510 mmol, 29%); TLC: ethyl acetate/*n*-hexanes = 6/2 (+ 1 % pyridine) R_f = 0.4; ^{1}H-NMR (400 MHz, $CDCl_3$, 25 °C): δ 8.44, 8.38 (m, 2H, 2x C(6)H); 7.39-7.20 (m, 18H, CH(ar)); 6.85-6.80 (m, 8H, CH(ar)); 5.74, 5.66 (2s, 2H, 2x C(1')H); 4.48 (s, 2H, 2x C(2')H); 4.40-4.30 (m, 4H, 2x C(3')H, 2x C(4')H); 3.79 (s, 12H, 4x OCH_3); 3.77-3.44 (m, 12H, (($CH_3)_2$**CH**$)_2$N, C(5'/5")H_2, 2x $POCH_2$); 2.59, 2.43 (2t, 4H, 2x **CH_2CN**); 2.27 (s, 6H, 2x NAc); 1.17-0.98 (m, 24H, 2x N(CH-**($CH_3)_2$**$)_2$); 0.92, 0.91 (2s, 18H, Si-tBu, TBDMS); 0.29 0.16 (2s, 12H, Si-$(CH_3)_2$, TBDMS)) ppm; ^{13}C-NMR (100 MHz, $CDCl_3$, 25 °C): δ 137.11 (d, $^{1}J_{CF}$ = 244.8 Hz, ^{13}C(5)F); 130.4 (CH(ar)); 128.3 (CH(ar)); 113.3 (CH(ar)); 91.6 (C(1')); 74.7 (C(2')); 70.2 (3')); 69.8 (C(4')); 60.4 (C(5'/5"); 57.0 (PO**C**H_2); 54.6 (OCH_3); 43.1 (C(3')H); 26.0 (Si-C$(CH_3)_3$, TBDMS); 24.8 (Si-(**C**$H_3)_2$); 24.1 (N(CH-**($CH_3)_2$**$)_2$); 19.8 (CH_2CN)**;** -4.97, -5.72 (Si-(**C**$H_3)_2$, TBDMS) ppm;^{19}F-NMR (375 MHz, $CDCl_3$, 25 °C): δ -166.18, -166.87 ppm; ^{31}P-NMR (162 MHz,

CDCl$_3$, 25 °C): δ 150.96, 148.39 ppm. ESI-MS: calculated for $^{13}CC_{46}H_{63}FN_5O_9PSi$ $[M+H]^+$: 921.4150, found 921.4202 $[M+H]^+$.

Synthesis of [5-^{19}F, 5-^{13}C] pyrimdine modified RNAs

Standard 2'-*O*-TBDMS RNA phosphoramidites (rA^{Ac}, rC^{Ac}, rG^{Ac} and rU, *Chemgenes*, USA) were used in combination with in-house synthesized [6-D, 5-^{13}C, 5-^{19}F] uridine and [5-^{19}F, 5-^{13}C]-modified pyrimidine RNA phosphoramidites. Controlled pore glass (CPG) RNA solid support (1000 Å pore size, *ChemGenes*, USA) with an average loading of 40 µmol g^{-1} was used to synthesize the RNAs on an ABI 391 PCR Mate using a self-written RNA synthesis cycle. Amidite (0.1 M) and activator (5-benzylthio-*1H*-tetrazole, 0.25 M) solutions were dried over freshly activated molecular sieves (3 Å) for at least 48 hours. The following reagent mixtures were used: *Cap A*: acetic anhydride/lutidine/tetrahydrofuran 1/1/8, v/v/v. *Cap B*: tetrahydrofuran/N-methylimidazole 86/16 v/v. *Oxidation solution*: 500 mg iodine dissolved in a mixture of 70 mL THF, 20 mL pyridine and 10 mL water. *Detritylation solution*: 4% dichloroacetic acid in anhydrous toluene. After complete RNA synthesis, the solid support was dried in 1h in high vacuum. *Standard alkaline deprotection*: 650 µL aq. methylamine solution (40 %) and 650 µL aq. ammonia solution (28-30 %) were added to the solid support. The reaction tube was shaken vigorously and incubated at 37°C for 6h. The solid support was pelleted via centrifugation and the supernatant was transferred to a 10 mL round bottom flask. The remaining solid support was washed three times with a mixture of THF/water (1/1), the liquid phases were combined with the first filtrate and evaporated to dryness. The residual white precipitate was dried in high vacuum for at least 1h. *2'-O-TBDMS deprotection:* The residue from the previous step was dissolved in 300 µL anhydrous dimethylsulfoxide, and triethylamine trihydrofluoride was added (50 eq. per TBDMS group) and the deprotection mixture incubated at 37°C for at least 16h. Then, the deprotection mixture was quenched with 2 mL quenching buffer (*GlenResearch*, USA) and directly applied to a HiPrep 26/10 desalting column (*GE Healthcare*, Austria) using a ÄKTA start system (*GE Healthcare*, Austria). The crude RNA was eluted using HPLC grade water and the RNA containing fractions (UV detection at 254 nm) were collected in a 50 mL round bottom flask. After evaporation, the crude RNA was dissolved in 1 mL HPLC grade water and transferred to a 1.5 mL reaction tube. The crude RNA was stored at -20°C. The quality of the crude RNAs was checked via anion exchange chromatography on an analytical Dionex DNAPac PA-200 column (4x250 mm; *Eluent A*: 25 mM Tris.HCl, 6 M urea, pH 8.0; *Eluent B*: 25 mM Tris.HCl, 500 mM sodium perchlorate, 6 M urea, pH 8.0) and at elevated temperature (80 °C). Purification of the RNA sequences was achieved in a single run by applying the crude RNA on a preparative Dionex DNAPac PA-200 column (22x250 mm, eluents as before). The fractions containing the desired RNA were pooled and loaded on a C18 SepPak catridge (*Waters*, Austria) to remove HPLC buffer salts. The RNA sodium salt form was then eluted from the C18 column with water/acetonitrile (1/1, v/v), concentrated and transferred to a 1.5 ml reaction tube for concentration determination and mass spectrometric analysis. Sample concentrations were determined by measuring UV absorption at 260 nm on a NanoPhotometer (*Implen*).

LC-ESI mass spectrometry

All RNAs were analyzed on Finnigan LCQ Advantage MAX ion trap instrumentation connected to a Thermo Scientific UHPLC (components: Ultimate 3000 RS Pump, Ultimate 3000 RS Autosampler, Ultimate 3000 RS Column Compartment, Ultimate 3000 Diode Array Detector). RNA mass spectra were acquired in the negative-ion mode with a potential of -4 kV applied to the spray needle (capillary temperature: 270°C, capillary voltage: -23V). LC: 250 pmol RNA dissolved in 30 µl of 20 mM ethylenediaminetetraacetic acid (EDTA) solution; average injection volume: 30 µl; column: Waters xBridge C18 2.5µm column (1.0 × 50 mm) at 30°C; flow rate: 100 µL/min; Eluent A: 8.6 mM triethylamine (TEA), 100 mM 1,1,1,3,3,3-hexafluoroisopropanol in H_2O (pH 8.0); Eluent B: methanol; gradient: 0–100% B in A within 30 min; UV detection at 260/280 nm. The correct assembly of all RNAs used in this study was confirmed by the mass data.

NMR spectroscopy

RNA samples were lyophilized as sodium salts and dissolved in 280 µL NMR buffer (15 mM sodium phosphate, 25 mM NaCl, 0.1% NaN_3, pH 6.9) and transferred into restricted volume Shigemi tubes giving 0.2 to 1 mM sample concentrations. Experiments were run at 298 K unless otherwise stated. All NMR experiments were conducted on a Bruker 600 MHz Avance II+ NMR with a Prodigy TCI probe. The ^{13}C-detected, ^{19}F-^{13}C out-and-stay TROSY experiment is available from Arthanari Laboratory at the Dana Farber Cancer Institute (https://artlab.dana-farber.org/19f_13c_aromatictrosy.html). For the HIV TAR 2 RNA 12 the following parameters were used: spectral width in the indirect ^{19}F dimension was set to 10 ppm, and the spectral width in the direct ^{13}C dimension was set to 10 ppm. A total of 64 complex points was collected in the indirect ^{19}F dimension (acquisition time = 5.7 ms) and 1024 complex points were collected in the direct ^{13}C dimension (acquisition time = 340 ms). 32 scans were collected with a recycling delay of 1 s resulting in an experimental time of 0.5h. The carrier frequency was placed at 140 ppm in the ^{13}C dimension and in the ^{19}F dimension at -166.5 ppm. The $^{1}J_{CF}$ coupling constant was set to 230 Hz. For the 100% [6-D, 5-^{19}F, 5-^{13}C]-U labelled hHBV ε RNA **13** the following parameters were used: spectral width in the indirect ^{19}F dimension was set to 10 ppm, and the spectral width in the direct ^{13}C dimension was set to 10 ppm. A total of 64 complex points was collected in the indirect ^{19}F dimension (acquisition time = 5.7 ms) and 1024 complex points were collected in the direct ^{13}C dimension (acquisition time = 340 ms). 64 scans were collected with a recycling delay of 1 s resulting in an experimental time of 1h. The carrier frequency was placed at 139 ppm in the ^{13}C dimension and in the ^{19}F dimension at -165 ppm. The $^{1}J_{CF}$ coupling constant was again set to 230 Hz. For the 25% [6-D, 5-^{19}F, 5-^{13}C]-U labelled hHBV ε RNA **13** the number of scans was increased to 288 resulting in an experimental time of 10h. For the 49nt [5-^{19}F, 5-^{13}C]-C *metK* SAM VI aptamer **14** the following parameters were used: spectral width in the indirect ^{19}F dimension was set to 10 ppm, and the spectral width in the direct ^{13}C dimension was set to 15 ppm. A total of 64 complex points was collected in the indirect ^{19}F dimension (acquisition time = 5.7 ms) and 1024 complex points were collected in the direct ^{13}C dimension (acquisition time = 226 ms). 64 scans were collected with a recycling delay of 1 s resulting in an experimental time of 1h. The carrier frequency was placed at 136 ppm in the ^{13}C dimension and in the ^{19}F dimension at -165 ppm. The $^{1}J_{CF}$ coupling constant was set to 245 Hz. For the 59nt [5-^{19}F, 5-^{13}C]-C and -U labeled pre-miR 21 **15** the following parameters were used: spectral width in the indirect ^{19}F dimension was set to 8 ppm, and the spectral width in the direct ^{13}C dimension was set to 10 ppm. A total of 64 complex points was collected in the indirect ^{19}F dimension (acquisition time = 7 ms) and 1024 complex points were collected in the direct ^{13}C dimension (acquisition time = 339 ms). 96 scans were collected with a recycling delay of 1 s resulting in an experimental time of 1.75h. The carrier frequency was placed at 137 ppm in the ^{13}C dimension and in the ^{19}F dimension at -165 ppm. The $^{1}J_{CF}$ coupling constant was set to 245 Hz. The apical stem loop sub-segment of pre-miR 21 **15a** was acquired with the following parameters: spectral width in the indirect ^{19}F dimension was set to 8 ppm, and the spectral width in the direct ^{13}C dimension was set to 20 ppm. A total of 80 complex points was collected in the indirect ^{19}F dimension (acquisition time = 8.9 ms) and 1024 complex points were collected in the direct ^{13}C dimension (acquisition time = 170 ms). 32 scans were collected with a

recycling delay of 1 s resulting in an experimental time of 1h. The carrier frequency was placed at 137 ppm in the ^{13}C dimension and in the ^{19}F dimension at -165 ppm. The $^{1}J_{CF}$ coupling constant was set to 245 Hz. All datasets were processed with Topspin 4.0.6 (Bruker Biospin).

UV melting curve analysis

The RNAs were lyophilized and then dissolved in 300 µL melting curve buffer (10 mM sodium phosphate, pH 7.0, 150 mM NaCl) to give RNA concentrations of 2 and 5 µM, respectively. Absorbance versus temperature profiles were recorded at 250 nm and 260 nm on a Cary-100 spectrophotometer equipped with a multiple cell holder and a Peltier temperature-control device in 1 mm pathlength cuvettes. Data were collected for five heating-cooling cycles at a rate of 0.7 °C/minute. Melting transitions were essentially the same with respect to the two different wavelengths and heating-cooling cycles. Melting point temperatures are reported as mean value of the five measurements. The thermodynamic parameters of the monomolecular melting processes were obtained by plotting the association degree α versus temperature and fitting the experimental data in *KaleidaGraph* (Synergy Software) using the following equation:

$$\alpha = \frac{1}{1+e^{\frac{\Delta H^0 - T\Delta S^0}{RT}}}$$

with α association degree and R ideal gas constant to give the enthalpy ΔH^0 and the entropy ΔS^0 of the melting transition.

Results and discussion on the chemical solid phase synthesis of [5-^{19}F, 5-^{13}C]-pyrimidine modified RNA. With the building blocks **7** and **11** available their performance was checked in solid phase RNA synthesis. Phosphoramidites **7** and **11** were used in combination with RNA TBS phosphoramidites and were incorporated with a high coupling efficiency (> 98%). The standard deprotection procedure using aqueous methylamine and aqueous ammonia solutions (AMA) at an elevated temperature followed by treatment with triethylamine hydrogen fluoride in dimethylsulfoxide and quenching with a triethylammonium acetate buffer could be applied. We obtained high quality crude products for both the 30nt HIV TAR 2 RNA **12**, the 61nt hHBV ε RNA **13**, the SAM VI aptamer **14** and the 59nt and 29nt pre-miR 21 RNAs **15** and **15a** and after purification by anion exchange chromatography the correct sequence assembly was confirmed by mass spectrometry (**Supporting Table 1** and **Supporting Figure S1**). To summarize, the [5-^{19}F, 5-^{13}C]-pyrimidine phosphoramidites **7** and **11** could be used in our solid phase synthesis protocol giving the unique possibility to freely select the number and positioning of the modified residues within RNAs up to 60 nucleotides. The minimally invasive nature of the fluorine labeling was exemplarily shown for the hHBV ε RNA **13** by UV melting curve analysis (see a short discussion in the next Supporting Information section).

Results and discussion on the minimally invasive nature of fluorine labeling - Thermodynamic properties of the unmodified and [5-^{19}F, 5-^{13}C] uridine modified hHBV ε RNA from UV melting curves.

The influence of [5-^{19}F]-pyrimidine labels on the global stability of the RNA was already reported. Hennig and co-workers and also Puffer *et al.* reported a negligible effect on the global stability by the pyrimidine fluorination.[1] For example, for the HIV TAR-2 RNA **12** with all six uridines carrying a [5-^{19}F]-U label a ΔT_m of only 1°C was found. Here, we assessed the influence of the [5-^{19}F, 5-^{13}C]-uridine labels on the thermodynamic stability of the hHBV ε RNA by comparing the UV melting curves of a per-[5-^{19}F]-U modified 61nt hHBV RNA **13** and the unmodified counterpart. The melting points and thermodynamic parameters were identical within the error limits again confirming the minimal influence of [5-^{19}F]-pyrimidine substitutions on the global stability of RNA (**Supporting Figure S2**). The observation is in accordance with the results of Schwalbe and co-workers on the [2-^{19}F]-adenosine labeled guanine sensing riboswitch [2]. The global stability of the RNA very likely remains unchanged by a compensatory effect – the reduced hydrogen bonding stability by an increased acidity of the uridine imino proton [3] is counteracted by an increased base stacking interaction due to the fluorine substitution.[4]

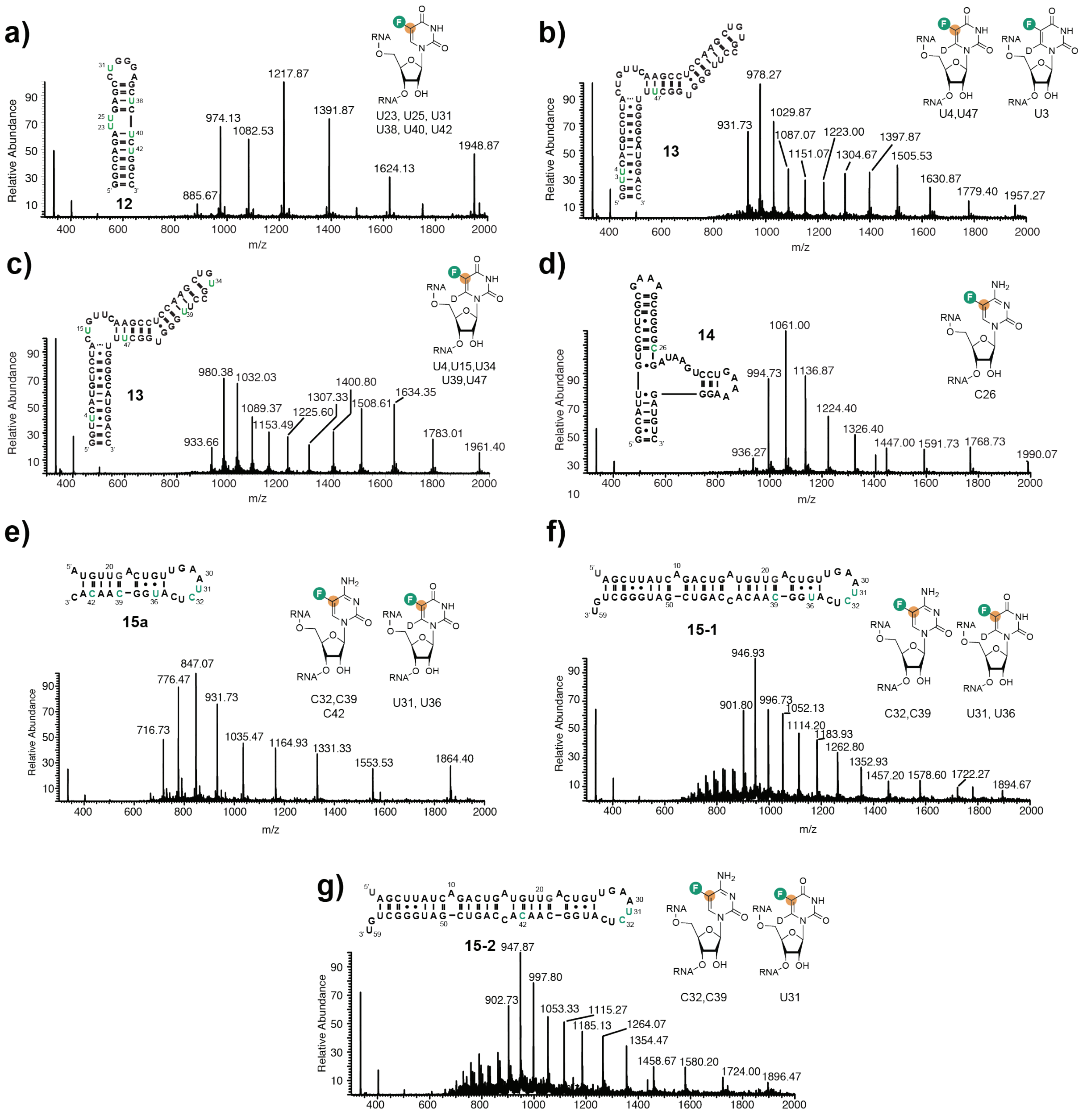

Supporting Figure S1. LC-ESI-MS spectra of RNAs. **a)** HIV TAR 2 RNA **12** with six [5-^{13}C, 5-^{19}F] uridine labels. **b)** hHBV ε RNA **13** with two [6-D, 5-^{13}C, 5-^{19}F]- and one [6-D, 5-^{19}F]-uridine labels. **c)** hHBV ε RNA **13** with five [6-D, 5-^{13}C, 5-^{19}F]-uridine labels. **d)** SAM VI **14** with one [5-^{13}C, 5-^{19}F]-cytidine label. **e)** pre-miR21 apical stem loop motif **15a** with three [5-^{13}C, 5-^{19}F]-cytidine and two [6-D, 5-^{13}C, 5-^{19}F]-uridine labels. **f)** pre-miR21 **15-1** with two [5-^{13}C, 5-^{19}F]-cytidine and two [6-D, 5-^{13}C, 5-^{19}F]-uridine labels. **f)** pre-miR21 **15-2** with two [5-^{13}C, 5-^{19}F]-cytidine and one [6-D, 5-^{13}C, 5-^{19}F]-uridine labels.

Supporting Table 1. Sequence information and mass spectrometric data for RNAs used in this study.

RNA	length	no. of [5-^{13}C, 5-^{19}F]-U/ [6-D, 5-^{13}C, 5-^{19}F-U]/ [6-D, 5-^{19}F]-U/ [5-^{13}C, 5-^{19}F]-C	molecular weight		
			calculated	found	Δmol.wt.
HIV TAR-2 **12**	30nt	6/0/0/0	9749.7	9750.8	1.1
hHBV ε **13**	61nt	0/2/1/0	19583.5	19584.0	0.5
hHBV ε RNA **13**	61nt	0/5/0/0	19624.5	19624.7	0.2
SAM VI **14**	49nt	0/0/0/1	15982.5	15928.5	0.0
Pre-miR 21 **15a**	29nt	0/3/0/2	9326.4	9327.3	0.9
Pre-miR 21 **15-1**	59nt	0/2/0/2	18976.1	18975.5	0.6
Pre-miR 21 **15-2**	59nt	0/1/0/2	18956.2	18956.2	0.0

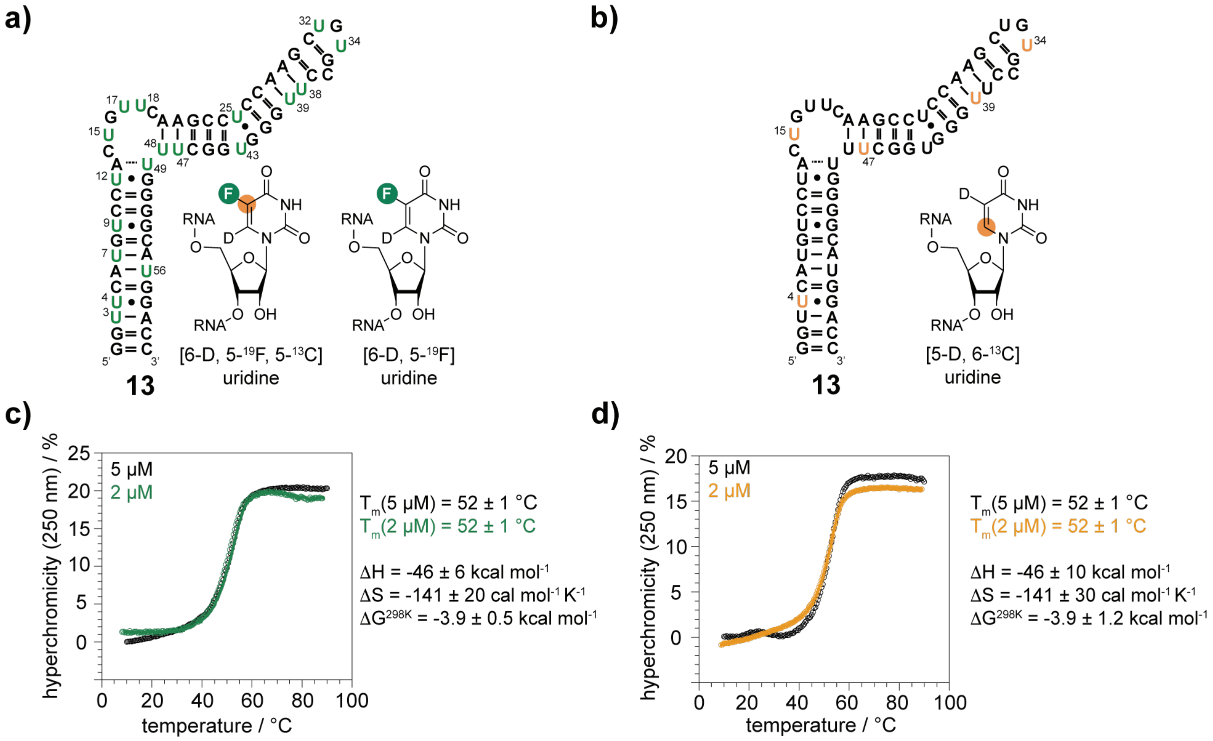

Supporting Figure S2. UV melting curves of [6-D, 5-^{13}C, 5-^{19}F]- and [6-D,5-^{19}F] uridine and [5-D, 6-^{13}C]-uridine labeled hHBV ε RNA **9**. **a)** Secondary structure of hHBV ε RNA **9** with fluorinated uridines highlighted in green. **b)** Secondary structure of hHBV ε RNA **9** with ^{13}C- and ^{2}H-labeled uridines highlighted in orange. **c)** UV melting curves at 2 and 5 µM concentration of the fluorinated RNA shown in a. The melting points are concentration independent indicative for a monomolecular fold. The thermodynamic parameters are given. **d)** UV melting curves at 2 and 5 µM concentration of the ^{13}C- and ^{2}H-labeled RNA shown in b. The melting points are concentration independent indicative for a monomolecular fold. The thermodynamic parameters are given.

Supporting Table 2. Labeling strategy to assign all 18 uridine resonances of hHBV ε RNA. The X marks the labeling position with 25% [6-D,5-^{13}C, 5-^{19}F] uridines, all other uridines in the sequence are [6-D, 5-^{19}F] uridine labeled.

**sample/U**	3	4	7	9	12	15	17	18	25	32	34	38	39	43	47	48	49	56
1	X	X	X									X				X	X	X
2			X	X	X					X	X	X	X	X		X		
3			X		X	X	X	X					X	X			X	
4								X	X	X		X		X	X			X
5		X				X					X		X		X			
**assigned by**	1	1+5	1+2+3	2	2+3	3+5	3	3+4	4	2+4	2+5	1+2+4	2+3+5	2+3+4	4+5	1+2	1+3	1+4

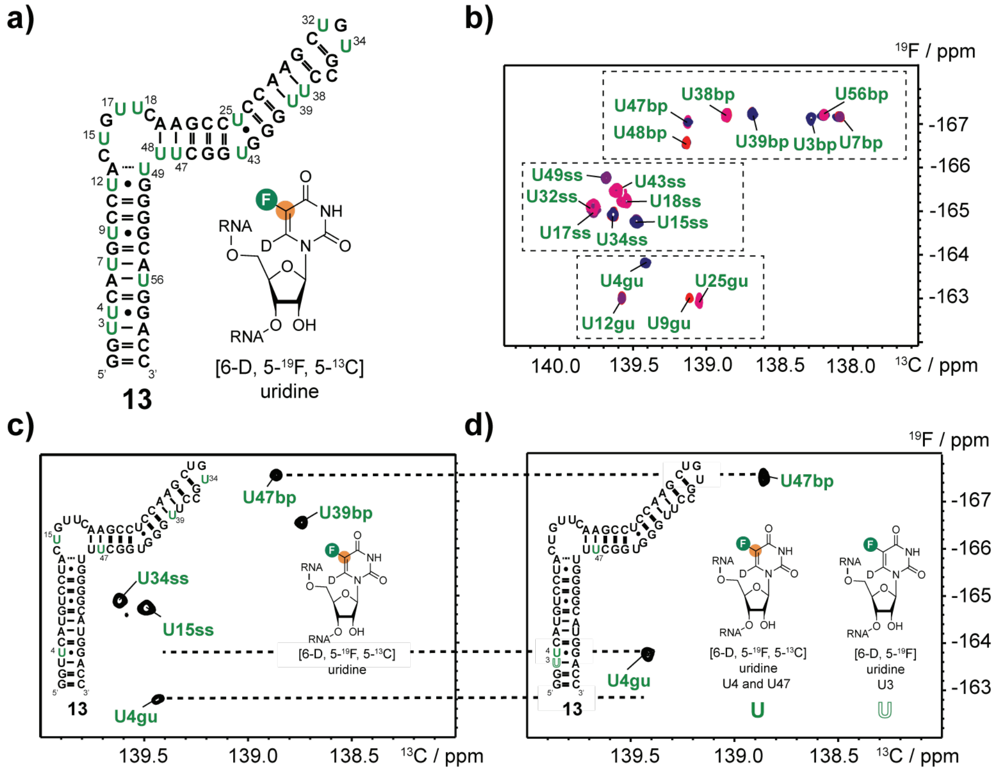

Supporting Figure S3. a) Secondary structure of the hHBV ε RNA with the uridines highlighted in green. The inset shows the [6-D, 5-^{19}F, 5-^{13}C] uridine label. **b)** Overlay of the five ^{13}C detected, out and stay ^{19}F-^{13}C TROSY spectra at 25°C of [6-D, 5-^{19}F, 5-^{13}C]/ [6-D, 5-^{19}F] uridine labeled RNAs **13** for assignments purposes. All resonances are shown with assignment. **c)** ^{13}C detected, out and stay ^{19}F-^{13}C TROSY spectrum at 25°C of [6-D, 5-^{19}F, 5-^{13}C] U4, U15, U34, U39 and U47 labeled RNA **13**. **d)** ^{13}C detected, out and stay ^{19}F-^{13}C TROSY spectrum at 25°C of [6-D, 5-^{19}F, 5-^{13}C]-U4 and U47 and of [6-D, 5-^{19}F]-U3 labeled RNA **13**. The shift change of U4 induced by its neighboring U3 (not fluorinated in c, fluorinated in d) is indicated by dashed lines.

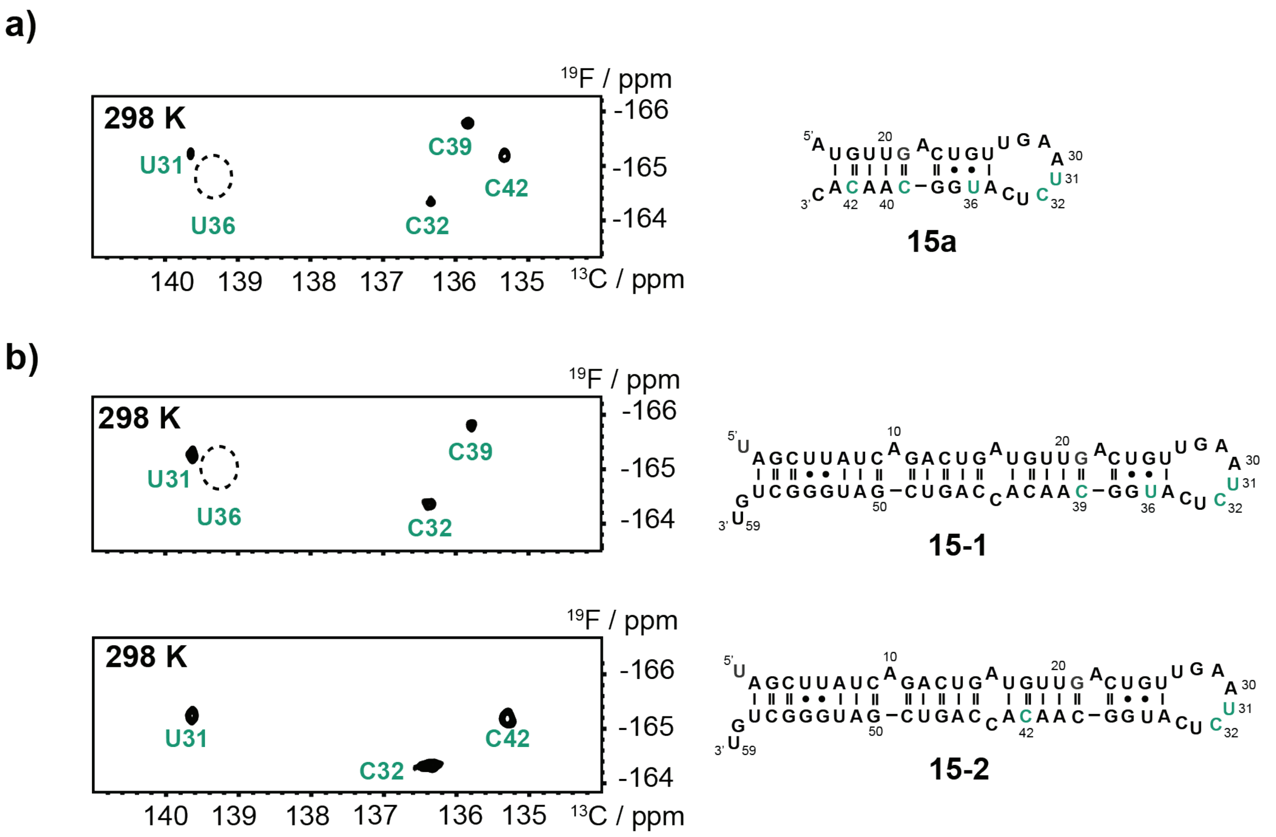

Supporting Figure S4. Pre-miR 21 NMR data at 25°C. **a)** ^{1}H detected, out and back ^{1}H-^{13}C TROSY at 25°C of **15a**. **b)** ^{1}H detected, out and back ^{1}H-^{13}C TROSY at 25°C of **15-1**. **c)** ^{1}H detected, out and back ^{1}H-^{13}C TROSY at 25°C of **15-2**. The U36 resonance , which is broadened beyomd detection is highlighted with a dashed circle.

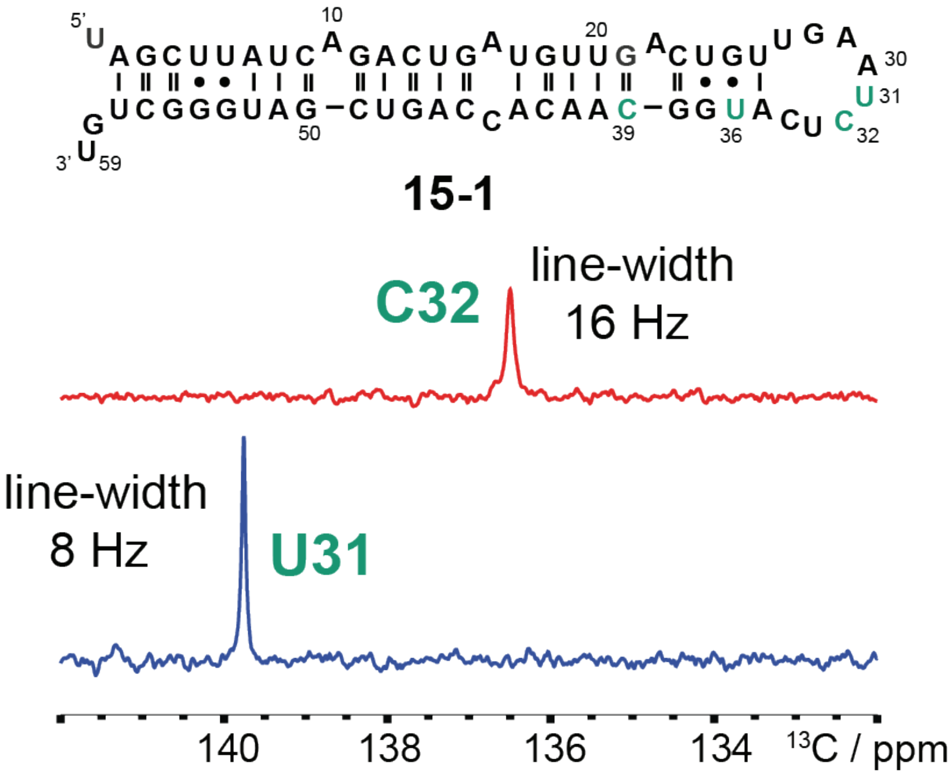

Supporting Figure S5. Line-width comparison of [6-D, 5-^{13}C, 5-^{19}F]-U31 and [5-^{13}C, 5-^{19}F]-C32 of the pre-miR 21 **15-1.**

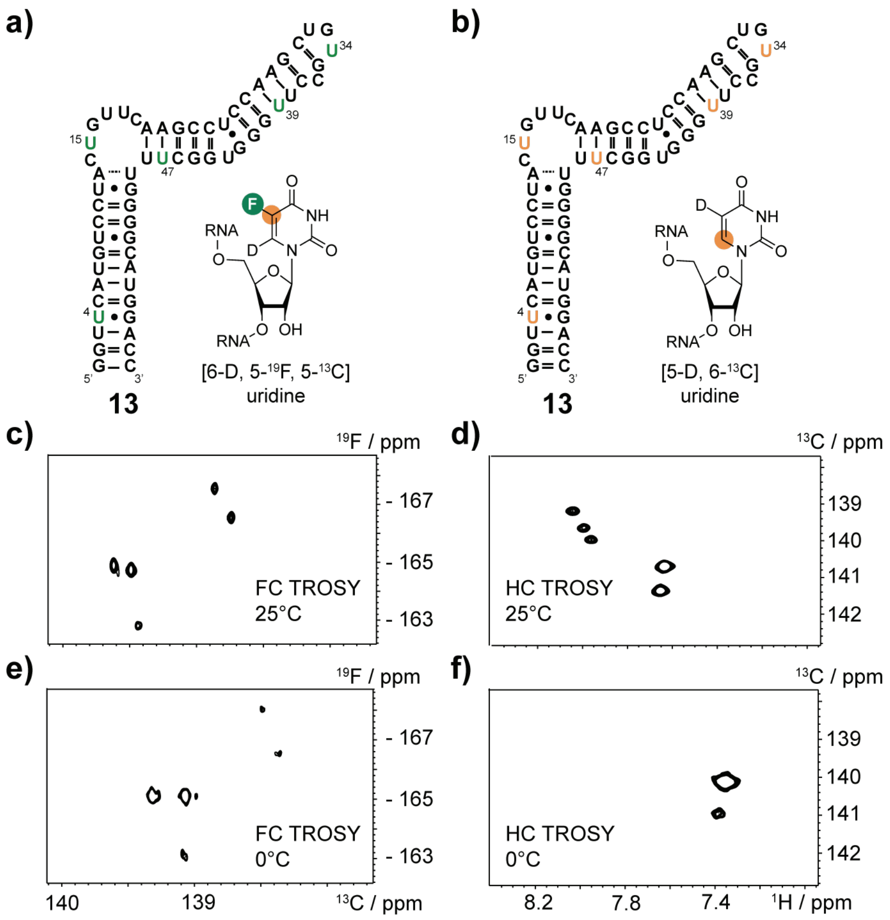

Supporting Figure S6. a) Secondary structure of the hHBV ε RNA with the [6-D, 5-^{19}F, 5-^{13}C] uridine labels highlighted in green. The inset shows the [6-D, 5-^{19}F, 5-^{13}C] uridine label. **b)** Secondary structure of the hHBV ε RNA with the [6-^{13}C , 5-D] uridine labels highlighted in orange. The inset shows the [6-^{13}C, 5-D] uridine label. **c)** ^{13}C detected, out and stay ^{19}F-^{13}C TROSY at 25°C of [6-D, 5-^{19}F, 5-^{13}C] uridine labeled RNA **13**. **d)** ^{1}H detected, out and back ^{1}H-^{13}C TROSY at 25°C of [6-^{13}C , 5-D] uridine labeled RNA **13**. **e)** ^{13}C detected, out and stay ^{19}F-^{13}C TROSY at 0°C of [6-D, 5-^{19}F, 5-^{13}C] uridine labeled RNA **9**. **f)** ^{1}H detected, out and back ^{1}H-^{13}C TROSY at 0°C of [6-^{13}C , 5-D] uridine labeled RNA **13**.

References

[1] aB. Puffer, C. Kreutz, U. Rieder, M.-O. Ebert, R. Konrat, R. Micura, *Nucleic Acids Research* **2009**, *37*, 7728-7740; bM. Hennig, L. G. Scott, E. Sperling, W. Bermel, J. R. Williamson, *Journal of the American Chemical Society* **2007**, *129*, 14911-14921.

[2] F. Sochor, R. Silvers, D. Müller, C. Richter, B. Fürtig, H. Schwalbe, *Journal of Biomolecular NMR* **2016**, *64*, 63-74.

[3] aA. D. Broom, V. Amarnath, R. Vince, J. Brownell, *Biochimica et Biophysica Acta (BBA) - Nucleic Acids and Protein Synthesis* **1979**, *563*, 508-517; bK. Mioduszewska, J. Dołżonek, D. Wyrzykowski, Ł. Kubik, P. Wiczling, C. Sikorska, M. Toński, Z. Kaczyński, P. Stepnowski, A. Białk-Bielińska, *TrAC Trends in Analytical Chemistry* **2017**, *97*, 283-296.

[4] M. R. Battaglia, A. D. Buckingham, J. H. Williams, *Chemical Physics Letters* **1981**, *78*, 421-423.
